# Supplementary material for: A sequential dual-site repetitive transcranial magnetic stimulation for major depressive disorder: A randomized clinical trial
Source: Cell Rep Med. 2025 Oct 1;6(10):102402. doi: 10.1016/j.xcrm.2025.102402 (PMC12629819; doi:10.1016/j.xcrm.2025.102402)

Cell Reports Medicine, Volume *6*

Supplemental information

A sequential dual-site repetitive transcranial magnetic stimulation for major depressive disorder: A randomized clinical trial

Yi-Jie Zhao, Shitong Xiang, Ruiqin Chen, Qiong Ding, Ruijie Geng, Yuan Wang, Yuanyuan Li, Haibin Li, Yichen Wang, Hailun Cui, Ying Huang, Jianfeng Feng, Wenjuan Liu, and Valerie Voon

Supplementary information for:

A Sequential Dual-Site Repetitive Transcranial Magnetic Stimulation for Major Depressive disorders: A Randomized Clinical Trial

Yi-Jie Zhao; Shitong Xiang; Ruiqin Chen; Qiong Ding; Ruijie Geng; Yuan Wang; Yuanyuan Li; Haibin Li; Yichen Wang; Hailun Cui; Ying Huang; Jianfeng Feng; Wenjuan Liu; Valerie Voon

Contents

Supplementary Tables 2

Table S1 Timeline for treatment and assessments. Related to Figure 1. 2

Table S2 Descriptive statistics of MADRS scores. Related to Figure 2. 2

Table S3 27 clusters identified by the WVCNA. Related to Figure 3. 3

Table S4 Statistics of Seed-Based Functional Connectivity (FC) with 27 clusters. Related to Figure 3. 3

Table S5 Statistics of Seed-Based Functional Connectivity (FC) Change Associated with MADRS improvement Ratio. Related to Figure 3. 4

Table S6 Functional Annotation Analysis Results. Related to Figure 3 and Figure S4. 5

Table S7 Statistics of Baseline Seed-Based Functional Connectivity (FC) Associated with MADRS improvement Ratio. Related to Figure 4. 6

Supplementary Figures 7

Figure S1. Results of secondary outcomes. Related to Figure 2. 7

Figure S2. Individual MADRS scores for long-term follow-up. Related to Figure 2. 7

Figure S3. High spatial similarity between the patterns of functional connectivity (FC) networks with the left dlPFC and dmPFC. Related to Figure 3. 8

Figure S4. Function annotation analysis results. Related to Figure 3 and Table S6 8

Supplementary Tables

Table S1 Timeline for treatment and assessments. Related to Figure 1.

	Baseline	Day 1	Day 2	Day 3	Day 4	Day 6	week-2 (Day 18)	week-4 (Day 32)
rTMS		•	•	•	•			
HAMD-6		•	•	•	•			
Clinician-rated scales	•				•		•	•
Self-rated scales	•				•		•	•
T1-weighted	•							
rsfMRI	•					•		

Abbreviations: rTMS, repetitive Transcranial Magnetic Stimulation; HAMD-6, Hamilton Depression Rating Scale (6-question version); rsfMRI, resting-state functional Magnetic Resonance Imaging.

Table S2 Descriptive statistics of MADRS scores. Related to Figure 2.

		Active group(n=26)				Sham-controlled group(n=26)			
		Mean	SD	95% CI		Mean	SD	95% CI	
				Lower Bound	Upper Bound			Lower Bound	Upper Bound
**MARDS score**	Baseline	27.42	3.82	25.92	28.92	26.15	4.25	24.55	27.75
	Treat end	16.04	6.99	13.34	18.74	20.85	5.97	18.55	23.15
	Two week	12.38	7.13	9.68	15.08	17.92	7.38	15.12	20.72
	Four week	13.50	8.04	10.4	16.6	21.31	8.38	18.11	24.51
**MARDS**	Treat end	0.42	0.25	0.32	0.51	0.19	0.25	0.09	0.29
	Two week	0.54	0.27	0.44	0.64	0.30	0.30	0.19	0.41
**improvement ratio**	Four week	0.51	0.27	0.41	0.61	0.17	0.33	0.04	0.30

*MARDS = Montgomery-Åsberg Depression Rating Scale, SD = Standard Deviation, 95% CI =95% Confidence Interval

Table S3 27 clusters identified by the WVCNA. Related to Figure 3.

Cluster label	Peak Point in MNI Space			Voxel Size	Brain Region Label
	x	y	z		
Cluster1	42	-17	51	407	Right pre/postcentral cortex
Cluster2	-53	7	29	213	Left inferior frontal cortex
Cluster3	48	-33	45	188	Right supramarginal gyrus
Cluster4	46	8	34	144	Right inferior frontal cortex
Cluster5	-50	20	9	121	Left inferior frontal cortex
Cluster6	-36	-17	62	113	Left postcentral cortex
Cluster7	-1	6	63	112	Supplementary motor cortex
Cluster8	40	-47	47	101	Right inferior parietal cortex
Cluster9	-44	-39	51	98	Left inferior parietal cortex
Cluster10	25	-24	-17	93	Right amygdala/hippocampus
Cluster11	-4	14	48	90	Middle cingulate cortex
Cluster12	37	-7	60	85	Right superior frontal cortex
Cluster13	-37	-43	43	81	Left inferior parietal cortex
Cluster14	-34	4	58	68	Left dorsolateral prefrontal cortex
Cluster15	-35	51	22	58	Left middle frontal cortex
Cluster16	-16	-8	-16	56	Left amygdala/hippocampus
Cluster17	-32	-54	49	52	Left superior parietal cortex
Cluster18	-61	-48	30	48	Left supramarginal gyrus
Cluster19	5	-3	-8	43	Right ventral thalamus
Cluster20	-51	-22	-28	41	Left inferior temporal cortex
Cluster21	37	38	28	41	Right middle frontal cortex
Cluster22	-48	38	-6	31	Left lateral orbitofrontal cortex
Cluster23	36	15	-34	29	Right temporal pole
Cluster24	52	-16	-26	23	Right inferior temporal cortex
Cluster25	39	45	-11	23	Right lateral orbitofrontal cortex
Cluster26	39	53	7	21	Right middle frontal cortex
Cluster27	0	38	-8	20	Subgenual anterior cingulate cortex

Table S4 Statistics of Seed-Based Functional Connectivity (FC) with 27 clusters. Related to Figure 3.

Cluster label	dlPFC FC					dmPFC FC				
	FC Change (Active vs Sham)			Post vs pre (Active)		FC Change (Active vs Sham)			Post vs pre (Active)	
	t	*p*	*Cohen' d*	t	*p*	*t*	*p*	*Cohen'd*	*t*	*p*
Cluster1	**3.316**	**0.002**	**1.011**	1.614	0.107	1.381	0.174	0.421	0.437	0.662
Cluster2	**3.108**	**0.003**	**0.948**	1.802	0.072	1.999	0.052	0.610	0.572	0.568
Cluster3	**2.830**	**0.007**	**0.863**	1.364	0.173	1.870	0.068	0.570	0.438	0.662
**Cluster4**	**3.555**	**0.001**	**1.084**	**2.039**	**0.042**	1.842	0.072	0.562	0.743	0.458
**Cluster5**	**2.765**	**0.008**	**0.843**	**2.035**	**0.042**	**2.429**	**0.019**	**0.741**	1.478	0.140
Cluster6	**2.756**	**0.009**	**0.841**	1.462	0.144	1.126	0.266	0.344	0.252	0.801
Cluster7	**3.409**	**0.001**	**1.040**	1.714	0.087	1.811	0.077	0.552	0.535	0.593
Cluster8	**2.432**	**0.019**	**0.742**	0.656	0.512	1.637	0.109	0.499	0.452	0.651
Cluster9	**2.679**	**0.010**	**0.817**	1.625	0.104	**2.343**	**0.024**	**0.715**	1.029	0.304
**Cluster10**	**-2.395**	**0.021**	**-0.730**	-1.820	0.069	**2.472**	**0.017**	**-0.754**	**-2.752**	**0.006**
Cluster11	**3.150**	**0.003**	**0.961**	1.840	0.066	1.810	0.077	0.552	0.743	0.458
Cluster12	**2.859**	**0.007**	**0.872**	1.005	0.315	1.453	0.153	0.443	0.080	0.936
Cluster13	**2.809**	**0.007**	**0.857**	1.533	0.125	**2.035**	**0.048**	**0.621**	0.512	0.609
Cluster14	**2.398**	**0.021**	**0.731**	1.665	0.096	0.523	0.603	0.160	-0.447	0.655
**Cluster15**	**3.400**	**0.001**	**1.037**	**2.575**	**0.010**	1.360	0.181	0.415	1.163	0.245
**Cluster16**	**-2.971**	**0.005**	**-0.906**	**-2.080**	**0.038**	**4.061**	**0.000**	**-1.239**	**-2.777**	**0.006**
Cluster17	**2.797**	**0.008**	**0.853**	1.865	0.062	1.508	0.139	0.460	1.054	0.292
Cluster18	**3.010**	**0.004**	**0.918**	1.093	0.275	1.786	0.081	0.545	0.850	0.396
**Cluster19**	-1.982	0.054	-0.604	-0.941	0.347	**4.121**	**0.000**	**-1.257**	**-2.540**	**0.011**
**Cluster20**	**-3.371**	**0.002**	**-1.028**	**-2.996**	**0.003**	**3.066**	**0.004**	**-0.935**	**-2.126**	**0.034**
Cluster21	**3.162**	**0.003**	**0.964**	0.981	0.327	1.246	0.219	0.380	0.186	0.852
**Cluster22**	**3.056**	**0.004**	**0.932**	**2.451**	**0.014**	**2.151**	**0.037**	**0.656**	1.823	0.069
**Cluster23**	-1.810	0.077	-0.552	-1.640	0.101	**3.105**	**0.003**	**-0.947**	**-2.160**	**0.031**
**Cluster24**	**-2.893**	**0.006**	**-0.882**	**-2.344**	**0.019**	**2.077**	**0.044**	**-0.634**	-1.660	0.097
**Cluster25**	**3.790**	**0.000**	**1.156**	**1.965**	**0.050**	**2.162**	**0.036**	**0.659**	1.531	0.126
Cluster26	**2.344**	**0.024**	**0.715**	0.835	0.404	1.585	0.120	0.483	1.079	0.281
**Cluster27**	-0.782	0.438	-0.239	-0.398	0.691	**3.726**	**0.001**	**-1.136**	**-2.866**	**0.004**

Table S5 Statistics of Seed-Based Functional Connectivity (FC) Change Associated with MADRS improvement Ratio. Related to Figure 3.

FC	Treatment End		week-4	
	t	*p*	t	*p*
**dlPFC**				
**Right inferior frontal cortex (Cluster4)**	**0.410**	**0.041**	**0.545**	**0.008**
Left inferior frontal cortex (Cluster5)	**0.441**	**0.030**	0.185	0.224
Left middle frontal cortex (Cluster15)	0.044	0.428	0.007	0.488
Left amygdala/hippocampus (Cluster16)	0.235	0.833	-0.010	0.484
Left inferior temporal cortex (Cluster20)	-0.115	0.320	-0.085	0.364
**Left lateral orbitofrontal cortex (Cluster22)**	0.317	0.093	**0.448**	**0.027**
Right inferior temporal cortex (Cluster24)	0.363	0.936	0.333	0.918
**Right lateral orbitofrontal cortex (Cluster25)**	**0.454**	**0.025**	**0.403**	**0.043**
**dmPFC**				
**Right amygdala/hippocampus (Cluster10)**	-0.233	0.169	**-0.433**	**0.032**
**Left amygdala/hippocampus (Cluster16)**	**-0.501**	**0.015**	**-0.470**	**0.021**
Right ventral thalamus (Cluster19)	0.034	0.554	-0.077	0.377
Left inferior temporal cortex (Cluster20)	-0.034	0.445	0.007	0.511
**Right temporal pole (Cluster23)**	-0.307	0.101	**-0.397**	**0.046**
**Subgenual anterior cingulate cortex (Cluster27)**	**-0.491**	**0.016**	**-0.477**	**0.019**

Table S6 Functional Annotation Analysis Results. Related to Figure 3 and Figure S4.

Terms	dlPFC	dmPFC	Terms	dlPFC	dmPFC	Terms	dlPFC	dmPFC
acoustic	0.4166	0.3938	judgment	0.0875	0.1469	recollection	**0.0265**	0.0669
action	0.2364	0.4552	judgment_task	0.0941	0.1421	repetition	0.2807	0.5266
action_observation	0.3362	0.5212	language	**0.0453**	0.3231	repetition_suppression	0.3816	0.3544
adaptation	0.1699	0.4437	language_comprehension	0.3761	0.3469	response_inhibition	**0.0307**	0.4492
affective	0.6278	0.0815	language_network	0.1204	0.5433	response_selection	0.3765	0.3425
anger	0.3337	0.3224	learning_task	0.3973	0.0612	reward	0.2633	0.2452
anticipation	0.4365	0.096	lexical_decision	0.0524	0.3478	reward_anticipation	0.3974	**0.0412**
anxiety	0.4204	**0.0288**	listening	0.2162	0.35	rhythm	0.3272	0.294
anxiety_disorders	0.4324	**0.0188**	major_depression	**0.0321**	**0.0049**	saccades	0.1498	0.2548
arithmetic	0.1833	0.3755	matching_task	0.3523	0.1388	sad	0.5181	**0.0261**
arousal	0.4939	**0.0337**	memory	0.4776	0.0561	salience	0.2735	**0.0173**
attention	0.2816	0.0904	memory_encoding	0.2295	0.4235	schizophrenia	0.3129	0.0954
attention_deficit	0.1185	0.3236	memory_load	**0.0134**	0.2401	secondary_somatosensory	0.4243	0.3926
attention_network	0.5759	0.4296	memory_performance	0.5474	0.0733	selective_attention	0.3884	0.3652
attentional_control	0.1647	0.3551	memory_processes	0.1932	0.4113	self	0.3706	0.0806
auditory_visual	0.4875	0.458	memory_retrieval	**0.0386**	0.0508	self_referential	0.1318	**0.0256**
autism	0.4178	0.0736	memory_task	0.2477	0.0619	self_reported	0.3237	0.318
autobiographical_memory	0.0681	**0.0105**	memory_wm	0.2175	0.3869	semantic	0.0597	0.0976
awareness	0.51	0.1773	mental_imagery	0.3259	0.5073	semantic_information	0.0623	0.1532
bipolar_disorder	0.0994	0.3444	mild_cognitive	0.5394	0.095	semantic_knowledge	0.1295	0.0865
cognition	0.3378	0.1063	monetary_reward	0.4149	0.0782	semantic_memory	0.1367	0.1259
cognitive_control	0.1822	0.321	monitor	0.5109	0.3463	sensation	0.3792	0.3283
cognitive_emotional	0.3735	**0.0443**	mood	0.4313	**0.0262**	sensory_information	0.1588	0.4243
cognitive_functions	0.1413	0.2714	moral	0.1468	0.1341	sensory_modalities	0.2715	0.2341
cognitive_processes	0.171	0.1345	motion	0.291	0.4916	sentence_comprehension	0.0601	0.2292
cognitive_tasks	0.454	0.1774	motivation	0.4507	0.1311	sleep	0.0751	0.4091
communication	0.5419	0.2136	motor_control	0.4563	0.4414	social	0.1415	0.0521
compulsive_disorder	0.1825	0.1301	motor_imagery	0.3011	0.3859	social_cognition	0.3897	0.1223
conscious	0.3338	0.0655	motor_performance	0.3401	0.3135	social_interaction	0.2788	0.0505
control_network	**0.006**	0.4423	motor_response	0.3675	0.4239	somatosensory	0.5477	0.4855
control_processes	0.1258	0.4581	motor_task	0.3882	0.3715	spatial_attention	0.1833	0.4324
decision_making	0.2777	**0.0291**	moving	0.2388	0.4568	speech	0.2713	0.5336
decision_task	0.1169	0.2068	multisensory	0.4145	0.3892	speech_perception	0.4146	0.3998
depression	0.1825	**0.0027**	music	0.3856	0.2521	speech_production	0.4029	0.3824
detection_task	0.206	0.4975	naming	0.4524	0.413	speech_sounds	0.1519	0.2427
discrimination_task	0.5159	0.3533	navigation	0.1462	0.148	spoken	0.4012	0.3719
disgust	0.3786	0.064	negative_affect	0.0507	**0.0409**	stop_signal	0.4037	0.3739
emotion	0.2977	**0.035**	negative_emotional	0.2788	**0.0224**	stress	0.4413	**0.011**
emotion_regulation	**0.0099**	0.0522	neurocognitive	0.2117	0.0944	stroop	**0.0441**	0.3386
emotional_faces	0.4809	**0.0489**	neutral_faces	0.4661	0.0505	stroop_task	**0.0483**	0.2838
emotional_information	0.3957	0.3649	neutral_pictures	0.3069	0.0629	subsequent_memory	**0.043**	0.0588
emotional_responses	0.3132	**0.0116**	neutral_stimuli	0.495	0.0505	suppression	0.6572	0.2827
emotional_stimuli	0.4165	**0.0442**	nociceptive	0.4754	0.4081	sustained_attention	0.3997	0.2364
emotional_valence	0.2917	**0.0177**	nogo	0.2656	0.3983	switch	0.1022	0.3606
empathy	0.2933	0.251	object_recognition	0.4409	0.4283	switching	0.1449	0.3639
encoding_retrieval	0.1859	0.0891	obsessive_compulsive	0.2019	0.1361	syntactic	**0.0361**	0.3103
episode	0.138	0.3339	ongoing	0.2297	0.4546	tapping	0.2983	0.3681
episodic_memory	**0.0406**	0.0744	oral	0.3648	0.3302	target_detection	0.2801	0.4345
executive_control	0.1872	0.4485	orientation	0.3683	0.4154	term_memory	0.1708	0.3655
executive_function	0.1065	0.3223	pain	0.5092	0.1712	theory_mind	0.2058	0.1602
expectancy	0.2149	0.3832	passive_viewing	0.327	0.3181	threatening	0.431	**0.0414**
eye_movements	0.1813	0.3212	percept	0.4968	0.463	touch	0.4446	0.4123
face	0.2185	0.3023	perception	0.5388	0.6207	verbal	0.3101	0.4214
face_recognition	0.3556	0.3463	perceptual	0.4374	0.5938	verbal_fluency	0.254	0.4082
face_stimuli	0.2734	0.255	performance_task	**0.0452**	0.2143	verbal_working	0.231	0.3122
facial_expressions	0.4548	0.0837	personality	0.1787	0.0648	video	0.164	0.5155
fear	0.4775	**0.0209**	phonetic	0.1578	0.3488	visual_attention	0.2115	0.4579
fearful_faces	0.4864	**0.0259**	phonological	0.1257	0.4834	visual_auditory	0.2329	0.4588
finger_movements	0.3906	0.438	picture	0.2427	0.0679	visual_information	0.487	0.4631
fusiform_face	0.3126	0.3069	planning	0.3492	0.4515	visual_motion	0.2907	0.2696
gain	0.3693	0.103	pleasant	0.3125	0.2943	visual_perception	0.3171	0.2981
gambling	0.353	0.0509	pressure	0.3066	0.2701	visual_spatial	0.1302	0.296

goal_directed	0.5107	0.2013	primary_auditory	0.2607	0.246	visual_stimuli	0.2557	0.382
hallucinations	0.1948	0.4605	primary_sensorimotor	0.3532	0.404	visual_stream	0.3453	0.3381
hand_movements	0.4121	0.3937	primary_somatosensory	0.3676	0.3657	visuo_spatial	0.1107	0.2662
happy	0.3804	0.3637	primary_visual	0.3261	0.4118	visuospatial	0.2367	0.4917
hyperactivity_disorder	0.2748	0.0645	reactivity	0.3757	**0.028**	voice	0.1792	0.2297
illusion	0.2955	0.2719	reading	0.1101	0.5151	vulnerability	0.2302	**0.0048**
image	0.4905	0.1982	reasoning	**0.0369**	0.4119	word_recognition	0.3534	0.3324
impulsivity	0.1889	0.0958	recall	**0.0497**	**0.029**	working_memory	0.353	0.5122
incentive	0.2249	0.4252	recognition	**0.0393**	**0.0461**	written	0.0876	0.4169
inhibition	0.2744	0.4286	recognition_memory	0.2278	0.2034	recollection	**0.0265**	0.0669
inhibitory_control	**0.0245**	0.2914	recognition_task	0.451	0.1535	repetition	0.2807	0.5266

Table S7 Statistics of Baseline Seed-Based Functional Connectivity (FC) Associated with MADRS improvement Ratio. Related to Figure 4.

FC	Treatment End		week-4	
	t	*p*	t	*p*
**dlPFC**				
Right inferior frontal cortex (Cluster4)	-0.146	0.269	-0.317	0.087
**Left lateral orbitofrontal cortex (Cluster22)**	**-0.424**	**0.031**	**-0.479**	**0.016**
Right lateral orbitofrontal cortex (Cluster25)	-0.176	0.229	-0.269	0.126
**dmPFC**				
Right amygdala/hippocampus (Cluster10)	0.023	0.462	0.233	0.161
Left amygdala/hippocampus (Cluster16)	**0.391**	**0.044**	0.309	0.093
Right temporal pole (Cluster23)	0.151	0.263	**0.380**	**0.049**
**Subgenual anterior cingulate cortex (Cluster27)**	**0.403**	**0.039**	**0.440**	**0.026**

Supplementary Figures

Figure S1. Results of secondary outcomes. Related to Figure 2.

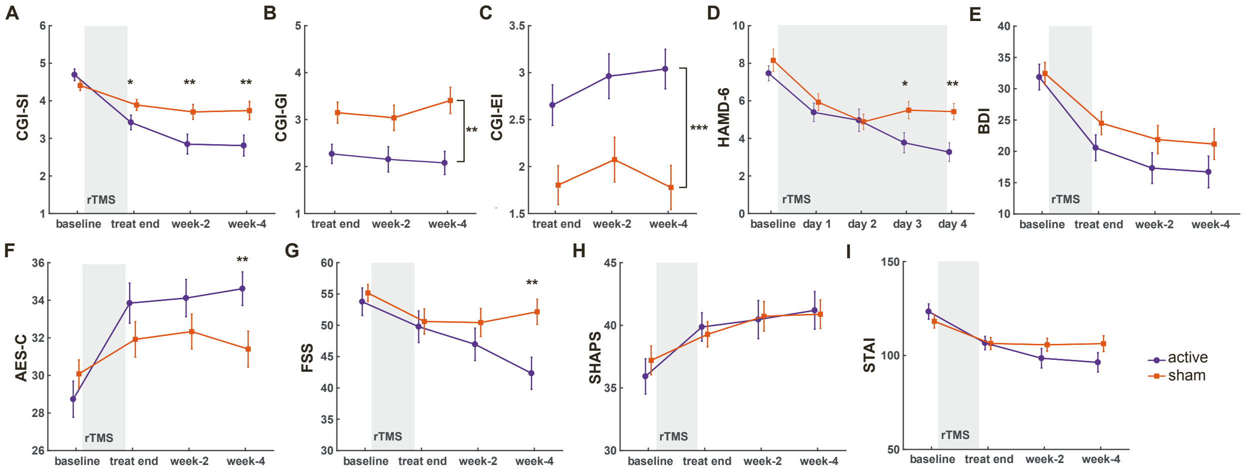

Figure S1. Results of secondary outcomes. CGI: the Clinical Global Impression; SI: severity of illness; GI: global improvement; EI: efficacy index; HAMD: Hamilton Depression Rating Scale; BDI: the Beck Depression Inventory; AES-C the Apathy Evaluation Scale – Clinician rated. (BDI), FSS: the Fatigue Severity Scale; SHAPS: the Snaith Hamilton Pleasure Scale. Data are represented as mean +/- SEM. Statistical analyses were conducted by mixed-measures ANOVA. Significance: $^{*}p$<.05; $^{**}p$<.01; $^{***}p$<.001.

Figure S2. Individual MADRS scores for long-term follow-up. Related to Figure 2.

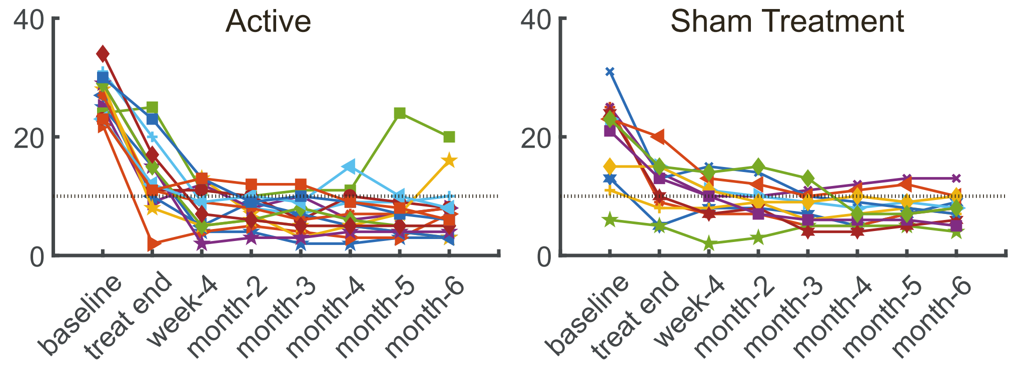

Figure S3. High spatial similarity between the patterns of functional connectivity (FC) networks with the left dlPFC and dmPFC. Related to Figure 3.

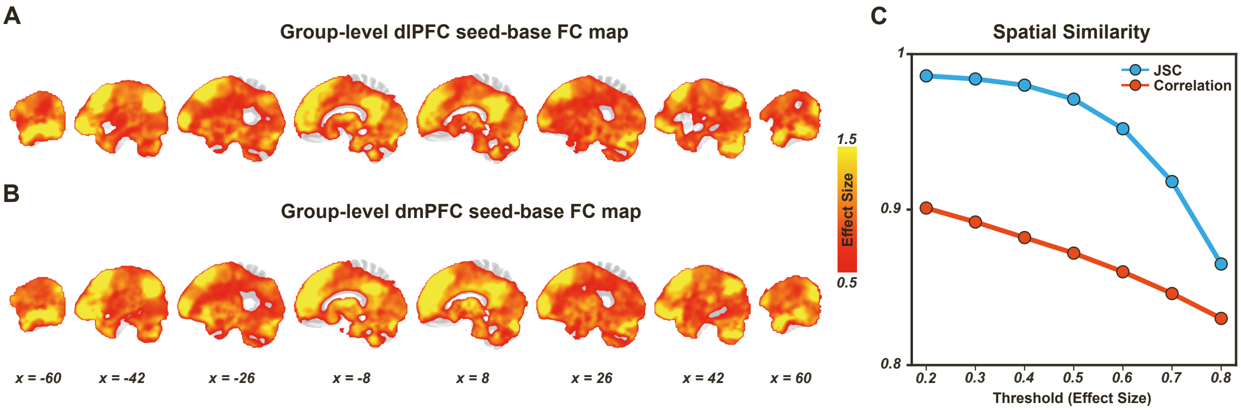

Figure S3. High spatial similarity between the patterns of functional connectivity (FC) networks with the left dlPFC and dmPFC. (a) The seed-based FC network targeting the left dlPFC. **(b)** The seed-based FC network targeting the dmPFC. **(c)** Statistical results of spatial similarity between above two FC networks under different thresholds.

Figure S4. Function annotation analysis results. Related to Figure 3 and Table S6.

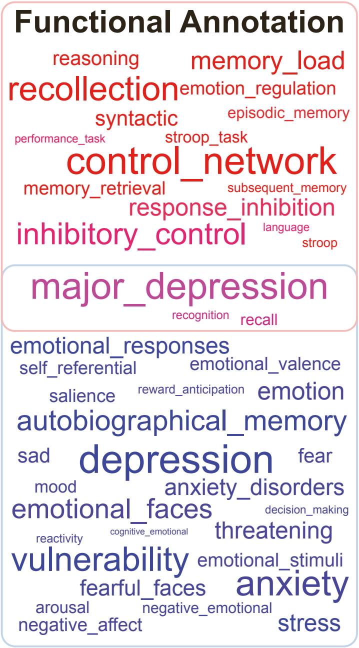

Supplement: Document S1. Figures S1–S4 and Tables S1–S7 [file mmc1.pdf]
