# Supplementary material for: A sequential dual-site repetitive transcranial magnetic stimulation for major depressive disorder: A randomized clinical trial
Source: Cell Rep Med. 2025 Oct 1;6(10):102402. doi: 10.1016/j.xcrm.2025.102402 (PMC12629819; doi:10.1016/j.xcrm.2025.102402)
Supplement: Data S1. Clinical trial-related documents — Data S1 include the clinical trials submission checklist, trial protocol and statistical analysis plan, and CONSORT checklist. This information is related to STAR Methods. [file mmc2.zip › trial protocol and statistical analysis plan.docx]

**Trial protocol and statistical analysis plan**

**Study type：**Interventional study

**Study design：**Parallel

**Objectives of Study：**To explore the effects of new dual-site transcranial magnetic stimulation therapy on the symptoms of patients with major depression and the underlying neural mechanisms.

**Inclusion criteria**

1. Male or female, age between 18 to 55 at the time of screening.
2. A primary diagnosis of unipolar major depressive disorder (MDD) by DSM-V.
3. Montgomery-Åsberg Depression Rating Scale (MADRS) ≥ 20 at the time of screening.
4. At least one antidepressant failure at adequate dose and duration.
5. On stable antidepressant medication regimen for at least 4 weeks prior to TMS therapy and agree to keep the medication status stable during the treatment and the 4-week follow-up period.
6. Informed consent form must be signed indicating that the participant understands the purpose of and procedures required for the study and is willing to participate in the study.

**Exclusion criteria：**

1. A current or prior diagnosis of a major psychiatric disorder (e.g., substance use disorder, psychosis, bipolar disorder, anorexia, obsessive compulsive disorder, schizophrenia) or MDD with psychotic features, bipolar or related disorders.
2. Acute suicidal or violent behaviour or history of suicide attempt within the last 4 weeks.
3. Other severe or unstable medical condition ((e.g., major surgery or stroke) or neurological diseases (e.g., Parkinson’ disease) or evidence of cognitive impairments that could interfere with the conduct of the current study, or pose any unacceptable risk to the participant.
4. History of other non-invasive neuromodulation treatment such as Electroconvulsive Therapy (ECT), Modified Electroconvulsive Therapy (MECT), repetitive Transcranial Magnetic Stimulation (rTMS), transcranial Electric Stimulation (tES) or Vagus Nerve Stimulation (VNS).
5. Presence of a specific contraindication for TMS/MRI (e.g., history of seizures, pacemaker or metallic implant).
6. Participant with claustrophobia
7. Participant who is pregnant, breast-feeding, or planning to become pregnant while enrolled in this study.
8. Unable to comply with study visit schedule and timeline.

**Primary outcome measure:** MADRS

**Secondary outcome measures:** CGI, BDI, STAI, SHAPS, AES, FSS, daily HAMD-6

**TMS：**

1. target sequence：20Hz dlPFC -> 5 mins -> 20Hz dmPFC
2. time：4 session/day, 4 successive days, inter-session-interval: at least 50 mins
3. new 20 Hz: 2s on, 4s off, repeat for 30 times, 1200 pulses in total
4. power：100% left resting motor threshold
5. sham：flip the coil of 90^o^

**Statistics analysis plan:**

1. **Continuous variables** (such as scores for questionnaires, etc.): repeated-measure ANOVA for improvement ratios. Two independent variables: group (active and sham) and time (baseline, day-4, week-2, week-4). Posthoc: LSD correction.
2. **Binary variables** (such as sex, medication status, etc.): chi-square analysis.
