# Supplementary material for: A sequential dual-site repetitive transcranial magnetic stimulation for major depressive disorder: A randomized clinical trial
Source: Cell Rep Med. 2025 Oct 1;6(10):102402. doi: 10.1016/j.xcrm.2025.102402 (PMC12629819; doi:10.1016/j.xcrm.2025.102402)
Supplement: Data S1. Clinical trial-related documents — Data S1 include the clinical trials submission checklist, trial protocol and statistical analysis plan, and CONSORT checklist. This information is related to STAR Methods. [file mmc2.zip › clinical-trials-submission-checklist-for-authors.pdf]

Clinical Trial Submission Checklist

If you have questions concerning these guidelines, please contact the Cell Press journal to which you are submitting your manuscript.

General

☒ Sex and Gender reporting according to the **SAGER**[1] guidelines:
Throughout the manuscript we ask authors to follow the “Sex and Gender Equity in Research” (SAGER) guidelines for reporting sex and gender.

Abstract

☒ Trial registration number.
☐ Clearly declared and reported primary and secondary outcomes.

Main text

☒ Completely defined and reported primary and secondary outcomes.

☒ Ethics approval statement and identity of the committee that approved the study.

☒ A statement confirming that informed consent was obtained from all participants (or their legal guardians), in writing and dated, before enrollment in the study. When applicable, studies involving minors should report collection of assent from the study participants in addition to the informed consent of their legal guardians.

☒ Reporting of harms/adverse effects, or a statement that no harms occurred.

☒ Sample size determination.
☒ Extended Author Contributions statement, including:

- The identity of the authors who performed (and if applicable, oversaw and replicated) statistical analyses;
- The identity of at least two authors who had unrestricted access to all data;
- The identity of the authors who prepared the first draft of the manuscript, reviewed it and edited it;
- A statement confirming that all authors agreed to submit the manuscript, read and approved the final draft and take full responsibility of its content, including the accuracy of the data and the fidelity of the trial to the registered protocol and its statistical analysis (if applicable).

☒ Funding sources.

☒ Conflict of Interest/Declaration of Interest statement.

Main figures

☒ CONSORT (Consolidated Standards of Reporting Trials) diagram in one of the main figures.

ASSOCIATED FILES AND FORMS

☒ CONSORT checklist (according to the CONSORT 2022 Harms guideline).

☒ Trial Protocol, including any amendments and the Statistical Analysis Plan (SAP).

[1] **https://www.equator-network.org/reporting-guidelines/sager-guidelines/**
