# Supplementary material for: A sequential dual-site repetitive transcranial magnetic stimulation for major depressive disorder: A randomized clinical trial
Source: Cell Rep Med. 2025 Oct 1;6(10):102402. doi: 10.1016/j.xcrm.2025.102402 (PMC12629819; doi:10.1016/j.xcrm.2025.102402)

Cell Reports Medicine

Article

A sequential dual-site repetitive transcranial magnetic stimulation for major depressive disorder: A randomized clinical trial

Graphical abstract

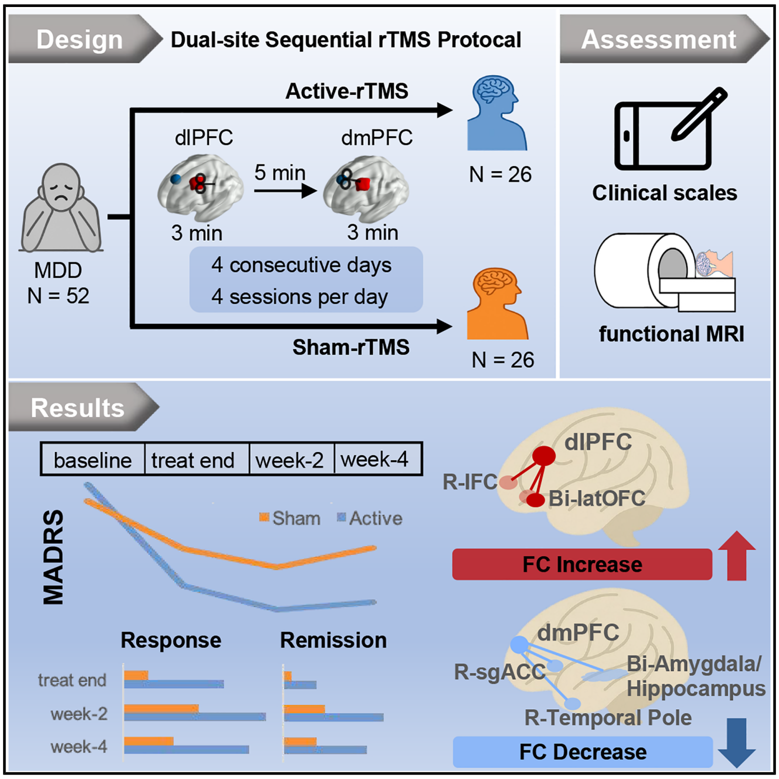

Authors

Yi-Jie Zhao, Shitong Xiang, Ruiqin Chen, ..., Jianfeng Feng, Wenjuan Liu, Valerie Voon

Correspondence

yj_zhao@tongji.edu.cn (Y.-J.Z.), wenjuan.liu@ufh.com.cn (W.L.), vv247@cam.ac.uk (V.V.)

In brief

Zhao et al. developed an accelerated dual-site rTMS protocol sequentially targeting the left dlPFC and dmPFC, demonstrating rapid and sustained antidepressant effects, possibly through normalizing functional connectivity in depression-related neural networks of the two targets. This cost-efficient intervention requires fewer pulses, no neuronavigation, and enables convenient patient engagement over 4 half-days.

Highlights

- The dlPFC-dmPFC dual-site accelerated rTMS protocol shows good treatment efficacy
- The rTMS protocol demonstrates rapid and sustained antidepressant effects
- Symptom improvement is linked to dlPFC-frontoparietal/ dmPFC-limbic neural networks

Zhao et al., 2025, Cell Reports Medicine 6, 102402
October 21, 2025 © 2025 The Authors. Published by Elsevier Inc.
https://doi.org/10.1016/j.xcrm.2025.102402

CellPress

Cell Reports Medicine

CellPress OPEN ACCESS

Article

A sequential dual-site repetitive transcranial magnetic stimulation for major depressive disorder: A randomized clinical trial

Yi-Jie Zhao,[1,2,*] Shitong Xiang,[2,3,4] Ruiqin Chen,[1,2] Qiong Ding,[5] Ruijie Geng,[6] Yuan Wang,[6] Yuanyuan Li,[6] Haibin Li,[6] Yichen Wang,[2,3,4] Hailun Cui,[5] Ying Huang,[1] Jianfeng Feng,[2,3,4] Wenjuan Liu,[6,7,*] and Valerie Voon[1,2,3,4,5,8,9,*]

[1]Clinical Research Center for Mental Disorders, Shanghai Pudong New Area Mental Health Center, School of Medicine, Tongji University, Shanghai, China

[2]Institute of Science and Technology for Brain-Inspired Intelligence, Fudan University, Shanghai, China

[3]Key Laboratory of Computational Neuroscience and Brain-Inspired Intelligence (Fudan University), Ministry of Education, Shanghai, China

[4]Zhangjiang Fudan International Innovation Center, Shanghai, China

[5]Department of Psychiatry, University of Cambridge, Cambridge, UK

[6]Department of Psychological Medicine, Zhongshan Hospital, Fudan University, Shanghai, China

[7]Department of Psychiatry, Shanghai United Family Pudong Hospital, Shanghai, China

[8]Senior author

[9]Lead contact

*Correspondence: yj_zhao@tongji.edu.cn (Y.-J.Z.), wenjuan.liu@ufh.com.cn (W.L.), vv247@cam.ac.uk (V.V.)

https://doi.org/10.1016/j.xcrm.2025.102402

SUMMARY

Repetitive transcranial magnetic stimulation (rTMS) is approved for major depressive disorder (MDD), but it is limited by variable efficacy. Here, we examine antidepressant effects of our sequential dorsolateral prefrontal cortex (dlPFC)-dorsomedial prefrontal cortex (dmPFC) accelerated rTMS protocol, which includes a 4-day treatment with 4 sessions per day. At week 4, the Montgomery-Åsberg Depression Rating Scale (MADRS) reduction is significantly larger in the active group, and critical, significant differences were apparent on day 4. For active and sham-controlled groups, respectively, response rates are 57.69% and 23.08%, and remission rates are 38.46% and 15.38%. Of responders, over 85% remain in remission over 6 months. Resting-state fMRI shows dissociable symptom improvement associated with increased dlPFC-frontoparietal and decreased dmPFC-amygdalo-subcallosal cingulate functional connectivity. We highlight a cost-efficient generalizable rTMS approach targeting differential networks in MDD, which shows rapid and sustained antidepressant effects with a relatively small number of pulses and minimal treatment duration. The study is registered with ChiCTR (ChiCTR2100046042).

INTRODUCTION

Depression is a major public health issue and a global leading cause of disability.[1] A significant proportion of patients remain resistant to conventional psychotherapy or medications.[2] Neuromodulation, particularly repetitive transcranial magnetic stimulation (rTMS) is effective for major depressive disorder (MDD). Stimulating the left dorsolateral prefrontal cortex (dlPFC) with high frequency daily over a period of 4–6 weeks is standard treatment in clinical use.[3] However, meta-analyses indicate only a small to medium effect size.[4,5] The standard protocol also has limitations for acute suicidal ideation because of the delayed response of rTMS. Daily administration over several weeks is also a barrier for patients with full-time jobs or live far from the hospital. For these reasons, there is a clinical need to shorten treatment duration, optimize targeting and improve efficacy of rTMS in MDD treatment.

The delivery schedule of rTMS can be accelerated by administering multiple stimulation sessions per day. This accelerated protocol is safe and may have comparable efficacy to the standard once daily schedule.[6,7] A significant advantage of accelerated rTMS is its capacity to deliver high-dose pulses within a condensed time frame, potentially resulting in a rapid-acting antidepressant effect.[6,8,9] Recently, the Stanford neuromodulation therapy (SNT), comprising 10 stimulation sessions targeting left dlPFC with 3 intermittent theta-burst stimulation (iTBS) in each session per day over 5 days, has demonstrated rapid early and sustained improvements in a moderate to severe MDD population with baseline average Montgomery-Åsberg Depression Rating Scale (MADRS) score higher than 33.[10,11] The inter-session-interval is important with 50–90 min demonstrating a cumulative effect on synaptic strengthening.[12,13] This constrains the number of sessions per day for an individual patient. In practice, the requirement for treatment spanning several consecutive

© 2025 The Authors. Published by Elsevier Inc.
This is an open access article under the CC BY license (http://creativecommons.org/licenses/by/4.0/).

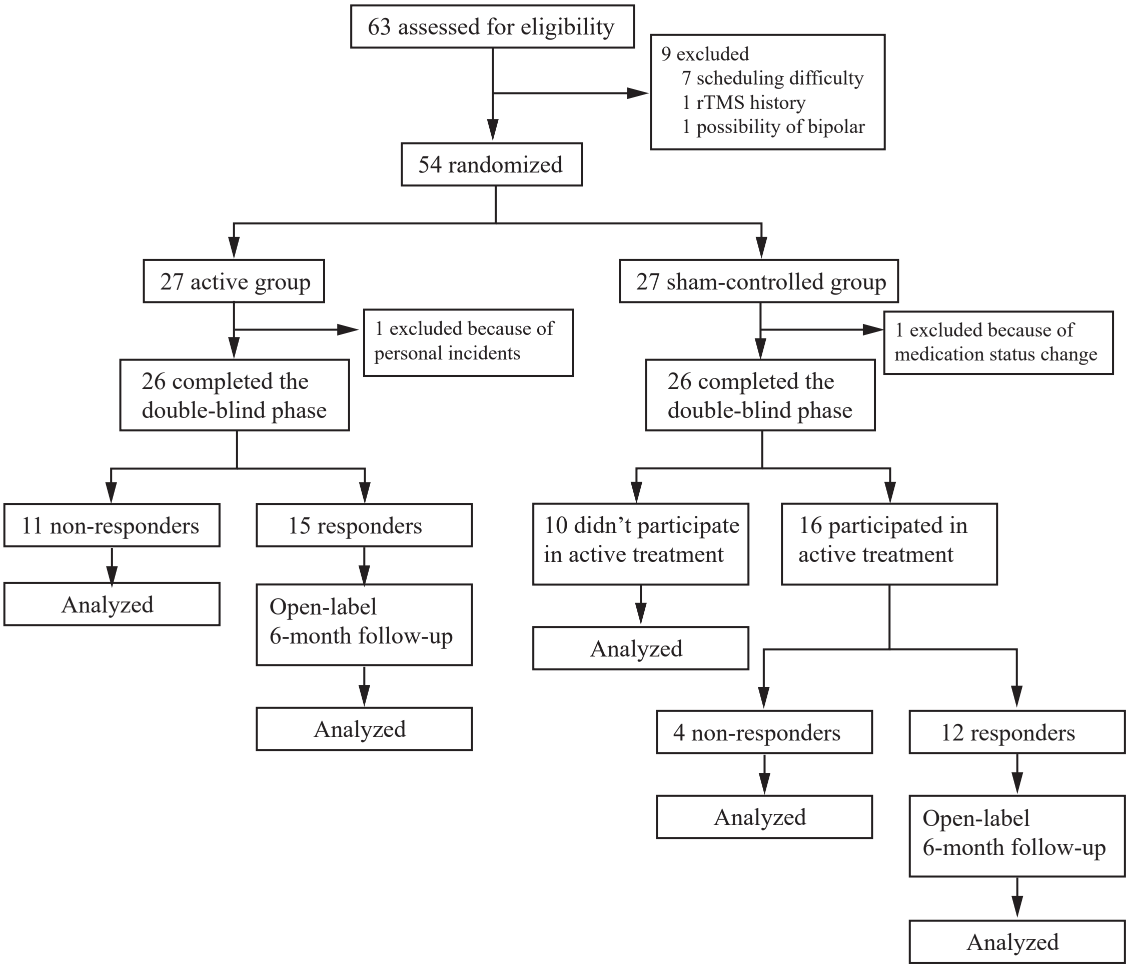

Figure 1. CONSORT flow diagram

days in a clinic limits the accessibility of outpatients with demanding work hours to this treatment.

MDD is characterized by heterogeneity in both underlying neural networks and phenotype.[14] Targeting multiple networks may facilitate the efficacy of accelerated rTMS. Beyond left dlPFC stimulation that shows moderate efficacy, the dorsomedial prefrontal cortex (dmPFC) has been identified as a potential superficial cortical candidate target. Convergent evidence has suggested dmPFC as a critical neutral substrate of depression. Gray matter volume is reduced in the dmPFC in MDD shown in a meta-analysis.[15] Baseline dmPFC metabolic activity distinguishes responders and non-responders following dlPFC-rTMS.[16] Moreover, inadvertent effect on dmPFC via deep brain stimulation may induce acute depressive symptoms reported in a case study of Parkinson's disease,[17] indicating a causal role of dmPFC in emotion processing. Functional neuroimaging identifies the dmPFC as a "dorsal nexus" in depression, given its convergent function of cognitive control, emotion regulation, and self-reflection.[18,19] Stimulating over dmPFC has shown potential efficacy in MDD[20–23] and critically shows efficacy in depression outcomes in two of four biotypes identified using resting state fMRI and anhedonia and anxiety symptoms. Evidence from double-blinded randomized controlled trials (RCTs) remains to be established.

Previous studies have indicated that sequential stimulation has potential to generate superior or more diverse effects on symptoms and brain networks. For example, low-frequency stimulation of the right dlPFC and high-frequency stimulation of the left dlPFC has demonstrated safety and efficacy in depression[24] with potentially higher odds ratios.[3] In addition, it has been proposed that the first stimulation may have a conditioning effect[25] if the two stimulated sites have overlapping networks.[26]

Here we developed a protocol to assess the effect of a dual-site approach stimulating left dlPFC and dmPFC sequentially in an accelerated manner with 16 sessions over 4 days versus sham stimulation in an outpatient MDD population. Subjects were scanned before and after treatment with the double-blind RCT followed for a 4-week assessment period. The placebo arm was then offered the dual stimulation protocol and responders were followed in an open-label manner up to 6 months. Using resting-state functional magnetic resonance imaging (rsfMRI), we first investigated if the two regions might have

Table 1. Demographic and clinical characteristics of the participants

	Active (n = 26)	Sham (n = 26)	Statistics t/χ^2	p
Sex (female/male)	12/14	19/7	3.914	0.048
Age (years, mean ± SD)	31.12 ± 7.14	33.15 ± 11.30	−0.778	0.440
Education (year, mean ± SD)	14.83 ± 3.56	14.81 ± 3.69	0.019	0.985
Duration (month, mean ± SD)	47.81 ± 40.63	65.58 ± 69.40	−1.127	0.265
Onset age (year, mean ± SD)	29.19 ± 8.84	27.92 ± 12.00	0.434	0.666
Medication (on/off)	26/0	25/1	1.020	1.000
SSRIs (on/off)	16/10	19/7	0.787	0.375
SNRIs(on/off)	8/18	9/17	0.087	0.768
Augmentation (on/off)	5/21	3/23	0.591	0.701
Benzodiazepines (on/off)	2/24	0/26	2.080	0.471
Baseline MADRS (mean ± SD)	27.42 ± 3.82	26.15 ± 4.25	1.133	0.263

MADRS, Montgomery-Asberg Depression Rating Scale; SSRIs, selective serotonin reuptake inhibitors; SNRIs, serotonin and norepinephrine reuptake inhibitors.

overlapping networks suggesting the potential for modulatory interactions between the two stimulated networks. We then asked how the resting-state neural network involving the two targets might dissociate and predict clinical outcomes.

RESULTS

Demographics and clinical characteristics

A graphic overview of the study is presented in Figure 1. A total of 63 participants were assessed for eligibility at Zhongshan hospital in Shanghai. Seven participants withdrew from the study because of scheduling difficulties. Two participants were excluded because they did not meet criteria in further assessment: one showed likely bipolar disorder; the other had a history of rTMS treatment failure. Additional two participants were excluded in the middle of the study: one participant encountered a major personal incident thus was unable to continue the treatment and the other participant arbitrarily changed medication doses. As a result, 52 participants (26 in each group) finished the study. This number meets the minimum requirements for this study according to the power analysis.

The demographic and clinical characteristics are summarized in Table 1. Chi-square analyses was used for sex, Selective serotonin reuptake inhibitors, SNRIs, augmentation, and benzodiazepines. Two-sample t tests (two-tailed) was used for other variables. Active and sham-controlled groups were not significantly different on baseline demographics (except for sex), medication status or baseline MADRS.

Analysis of study blinding and unblinding

Before unblinding at week 4, all participants were asked to guess whether they thought they were receiving active or sham rTMS treatment. A guess of active rTMS was made by 84.62% participants (22 out of 26) in the active group and 93.31% participants (24 out of 26) in the sham-controlled group (χ^2 = 0.046, p = 0.830). At the same time, we also asked the assessor to guess the group assignment of each participant before unblinding. The assessor guessed active rTMS of 14 (53.85%) and 15 (57.69%) participants in active and sham-controlled groups (χ^2 = 0.078, p = 0.780), respectively.

Safety

Adverse events are listed in Table 2. No severe adverse events occurred during the trial. "Discomfort at stimulation site" refers to uncomfortable sensations on the scalp or nearby muscles experienced during stimulation, while "headache/dizziness" indicates persistent discomfort after stimulation. The only adverse event that attained statistical significance was a higher rate of discomfort at the stimulation site reported in the active group compared to the sham-controlled group (χ^2 = 6.933, p = 0.008). This discomfort was more frequently reported during the dmPFC stimulation as this site is closer to the eyes. All adverse events were self-resolved and limited to previously reported adverse events by rTMS, especially in accelerated designs.[10,27]

Primary outcomes

Mixed-measures ANOVAs were used for statistical analyses for scores of clinical scales, with group as between-subject variables and MADRS scores as within subject variables. Post-hoc analyses were corrected by least significant difference. According to the demographic analysis, there was a sex difference between groups. Thus, in the following analyses, we put sex as a covariate. All mean values, standard deviations and 95% confidence intervals are listed in Table S2.

We first conducted a mixed-measures ANOVA with group as a between-subject variable and MADRS score (at baseline and week 4) as a within subject variable, to investigate whether the primary outcome measure (i.e., MADRS score at week 4) showed a significant improvement. The results showed a main effect of time (F(1,49) = 5.640, p = 0.022, η_p^2 = 0.103) indicating generally lower MADRS scores at week 4 and a significant group by time interaction (F(1,49) = 13.197, p = 0.001, η_p^2 = 0.212). The post-hoc analysis showed significant lower MADRS scores at week 4 in both groups (active: mean difference = 13.875, p < 0.001; sham: mean difference = 4.894, p = 0.006), as well as a lower MADRS scores in the active group compared to the sham-controlled group at week 4 (mean difference = 6.851, p = 0.005) but not at baseline (mean difference = 2.130, p = 0.056). The main effect of sex (as a covariate) was significant (F(1,49) = 6.088, p = 0.017, η_p^2 = 0.111), but no time by sex interaction effect was revealed (F(1,49) = 0.020, p = 0.887, η_p^2 = 0.000).

Table 2. Adverse events

	Active (n = 26)	Sham (n = 26)	Statistics t/χ^2	p
Fatigue	0 (0%)	2 (7.69%)	2.080	0.471
Discomfort at treatment site	10 (38.46%)	2 (7.69%)	6.933	0.008
Headache/dizziness	3 (11.54%)	4 (15.38%)	0.165	1
Nausea	1 (3.85%)	1 (3.85%)	–	–
Sleeping problems	1 (3.85%)	0 (0%)	1.020	1
Anxiety	0 (0%)	1 (3.85%)	1.020	1
Tinnitus	0 (0%)	0 (0%)	–	–
Seizure	0 (0%)	0 (0%)	–	–

Next, as we are also interested beyond week 4, we ran a mixed-measures ANOVA for 2 groups and 4 time points (baseline, day 4, week 2, and week 4) to investigate the clinical outcomes between two groups at different assessment time. Greater MADRS reduction was found over time in active compared to sham stimulation, reflected in a main effect of group (F(1,49) = 6.730, p = 0.012, η_p^2 = 0.121) and a group by time interaction (Figure 2A, F(3,47) = 5.125, p = 0.004, η_p^2 = 0.246). The post-hoc analysis showed no significant difference during the baseline assessment (mean difference = 2.130, p = 0.056) between two groups but significant differences at treatment end on day-4 (mean difference = 4.704, p = 0.016), week 2 (mean difference = 5.379, p = 0.014), and week 4 (mean difference = 6.851, p = 0.005). Neither main effect of sex (F(1,49) = 1.766, p = 0.190, η_p^2 = 0.035) or time by sex interaction were significant (F(3,47) = 1.229, p = 0.310, η_p^2 = 0.073). Similarly, MADRS reduction ratio (normalized to baseline) showed significant group by time interaction (Figure 2B, F(3,47) = 5.233, p = 0.003, η_p^2 = 0.250) and all group comparisons between follow-up time point was significant (ps < 0.004).

The rates of response and remission fluctuated at different follow-up time points due to the participants' diverse response trajectories (see Figures 1C and 1D for individual trajectories). At the endpoint (i.e., week 4), the response rate was 57.69% and 23.08% in active and sham-controlled groups, and the remission rate was 38.46% and 15.38% in active and sham-controlled groups, respectively. Response rates at treatment end and week 2 were 46.15%, 65.38%, and 11.54%, 34.62% in active and sham-controlled groups, respectively. Remission rates were 15.38%, 46.15%, and 3.85%, 19.23% in the active and sham-controlled group, respectively (Figures 2E and 2F).

Secondary outcomes

Mixed-measures ANOVAs with two groups as between-subject variables, scores as within-subject variables and sex as a covariant were conducted for secondary outcomes.

The CGI-SI had a significant interaction (Figure S1A, F(3,47) = 4.344, p = 0.009, η_p^2 = 0.217) and differences were found at week 2 (p = 0.014) and week 4 (p = 0.031) between two groups but not at baseline (p = 0.079) and day 4 (p = 0.072). Significant group differences were found for the CGI-GI (Figure S1B, F(1,49) = 6.364, p = 0.015, η_p^2 = 0.115) and CGI-EI (Figure S1C, F(1,49) = 15.094, p < 0.001, η_p^2 = 0.235) with no main effect of time or interaction. No effect of sex was revealed in these analyses. Note that these two scales were only assessed after treatment but not at baseline because they are used for treatment efficacy evaluation. Our double-blind clinical global impression (CGI) results highlight the efficacy of our protocol.

The daily rated Hamilton depression rating scale with only 6 questions (HAMD-6) showed an interaction effect (Figure S1D, F(4,46) = 3.268, p = 0.019, η_p^2 = 0.221) driven by significant lower scores at day 3 (p = 0.048) and day 4 (p = 0.006) in the active group compared to the sham-controlled group, but not at baseline (p = 0.888), day 1 (p = 0.729) or day 2 (p = 0.691). In addition, the post-hoc analysis revealed significant differences between day 4 and the first three time points (baseline: p < 0.001; day 1: p < 0.001, day 2: p = 0.001, day 3: p = 0.209) in the active group. In contrast, in the sham-controlled group, this comparison reached significance only between day 4 and baseline (p < 0.001, ps > 0.235 for the other three time points). These findings suggest that the active stimulation might exert a sustained effect over the course of treatment days, whereas such persistence of effect is not observed in the sham-controlled group.

Interestingly, for the self-reported Beck depression inventory (BDI) scores (Figure S1E), expect for the main effect of time (F(3,47) = 3.323, p = 0.028, η_p^2 = 0.157), no group (F(1,49) = 0.327, p = 0.570, η_p^2 = 0.007) or interaction (F(3,47) = 0.687, p = 0.565, η_p^2 = 0.042) effects were revealed, indicating the distinguished symptom evaluations of patients and clinicians.

The Apathy Evaluation Scale – Clinician rated (AES-C) showed a significant interaction (Figure S1F, F(3,47) = 6.266, p = 0.001, η_p^2 = 0.286), driven by significant difference at week 4 between the two groups (p = 0.033). Worthy mentioning, in the active group, all three time points after treatment showed decreased score compared to baseline (ps < 0.001), while in the sham-controlled group, scores were only reduced at week 2 (p = 0.035) but not at treatment end (p = 0.051) or week 4 (p = 0.143). These results may also indicate a non-lasting placebo effect.

The fatigue scale also showed a significant interaction effect of treatment (Figure S1G, F(3,47) = 3.666, p = 0.019, η_p^2 = 0.190), indicated by greater reduction at the week 4 time point between active and sham-controlled groups (p = 0.008).

No significant interaction was found for Snaith-Hamilton Pleasure Scale (SHAPS) (Figure S1H, F(3,47) = 0.244, p = 0.865, η_p^2 = 0.015) or State-Trait Anxiety Inventory (STAI) (Figure S1I, F(3,47) = 1.139, p = 0.343, η_p^2 = 0.068).

Sham treatment and follow-up

For participants in the sham-controlled group, after unblinding at the end of the week 4 assessment, they were offered an opportunity to receive active 4-day treatment with the same dual-site stimulation protocol. Sixteen participants chose to get this treatment. MADRS was assessed for these participants at the last day of this active treatment (13.75 ± 6.27) and also at week-4 (13.19 ± 8.22). Their MADRS scores at week-4 during the RCT were used as baseline (22.31 ± 9.09) for efficacy evaluation. We performed a mixed-measures ANOVA for the three time points and found a significant effect of time (F(2,14) = 32.984, p < 0.001, η_p^2 = 0.687). Treatment end (p < 0.001) and week-4

CellPress OPEN ACCESS

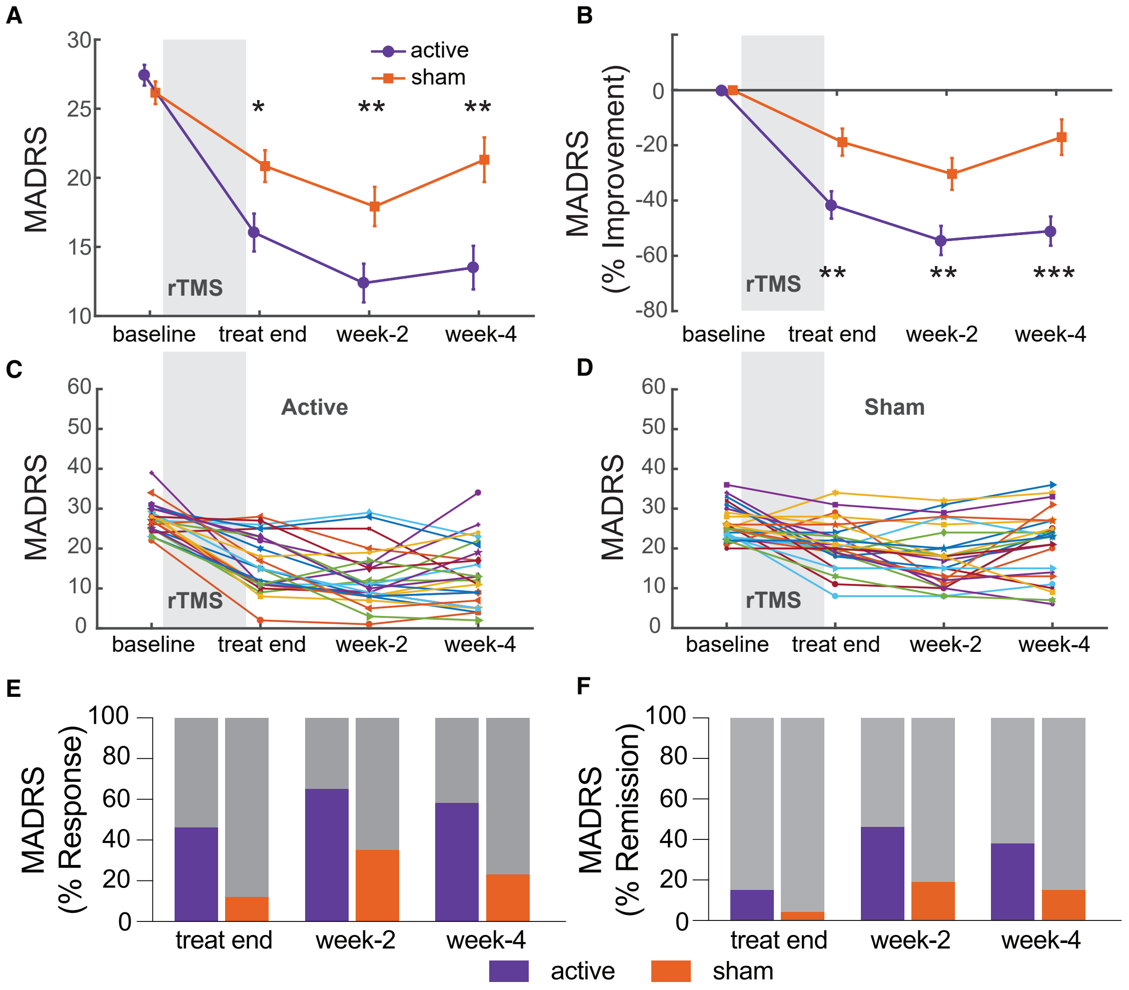

Figure 2. Clinical Outcomes

(A and B) Montgomery-Åsberg Depression Rating Scale (MADRS) scores (A) and its improvement ratio (normalized to baseline) (B) of participants in active and sham-controlled groups assessed at baseline, the last day of treatment, 2 weeks, and 4 weeks after treatment. Data are represented as mean ± SEM. Statistical analyses were conducted by mixed-measures ANOVA. Significance: $^*p < 0.05$, $^{**}p < 0.01$, and $^{***}p < 0.001$.

(C and D) Individual trajectories of MADRS scores for active and sham-controlled groups, respectively.

(E and F) Response (MADRS reduction $\geq$50%) rate and remission (MADRS $\leq$10) rate over time based on MADRS scores.

($p < 0.001$) assessment showed significant reduction compared to baseline but no difference was found between the two time points ($p = 0.580$). Response rate was 56.25% and remission rate was 43.75%. We would like to emphasize that a subset of participants in the sham-controlled group exhibited a placebo effect, introducing potential biases to the baseline we are utilizing. The average MADRS reduction is 9.39% in these participants compared to their RCT baseline. One participant even reached the criteria of remission before getting this treatment.

For all participants who met the response criteria at week 4, including the active group and the sham treatment group, we asked whether they were willing to have a monthly follow-up for up to 6 months after treatment. Twenty-eight participants took part in this long-term follow-up and only MADRS was assessed (Figure S2). The average (standard deviation) MADRS scores at 2–6 months was 8.32 (2.85), 7.25 (2.90), 7.14 (2.96), 7.57 (3.99), and 7.68 (3.74) and remission rates were 85.71% (24/28), 85.71% (24/28), 85.71% (24/28), 89.29% (25/28), and 89.29% (25/28), respectively. These results show a robust long-term efficacy of our protocol for treatment responders.

fMRI results

We first confirmed high spatial similarity between the patterns of FC networks of our dlPFC and dmPFC targets (correlations: $r > 0.8$, overlap: Jaccard index >0.8, Figure S3). Next, we identified clusters with clustering algorithms and asked which connections most contributed to the therapeutic effect (Figures 3A and 3B; Table S3). Twenty-seven clusters were initially identified according to the threshold: *Cohen's d* > 0.8. Then regions of interest (ROIs) were further restricted according to defined criteria. We show that FC between the dlPFC and the right inferior frontal cortex and bilateral lateral orbitofrontal cortex (latOFC) was

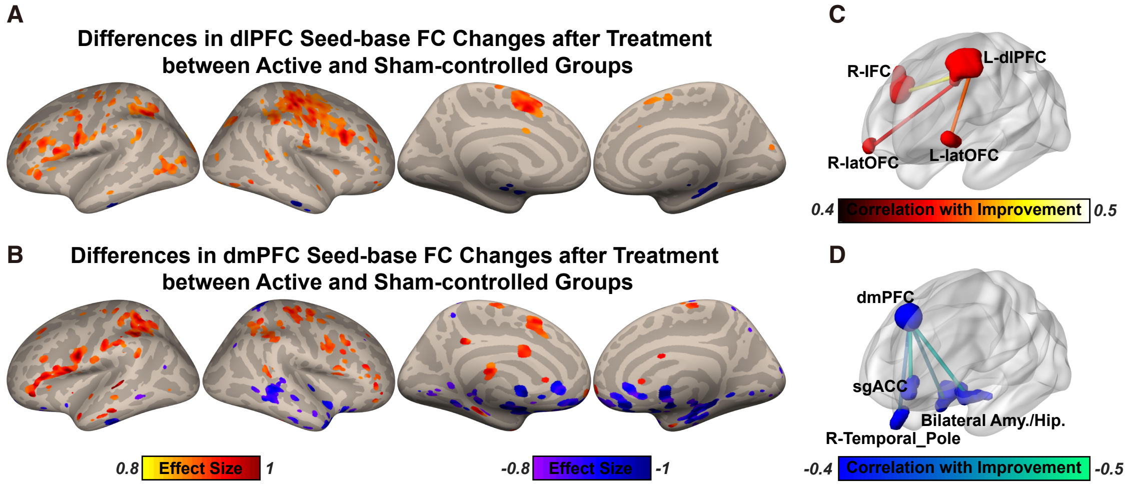

Figure 3. Neural circuits of the left dlPFC and dmPFC underlying the therapeutic effect of the dual-site rTMS treatment
(A and B) Voxel-wise alterative connections with the left dlPFC and dmPFC after the rTMS treatment, respectively.
(C and D) Associations between the long-term therapeutic effect and the cluster-wise alterative connections with the left dlPFC and dmPFC, respectively.

increased and associated with MADRS improvement ratio at week 4 (Figure 3C; Tables S4 and S5). In contrast, FC with dmPFC was decreased, including bilateral amygdala/hippocampus, the right temporal pole and the subgenual anterior cingulate cortex (sgACC) (Figure 3D; Tables S4 and S5). To further examine whether dlPFC and dmPFC show FC change patterns associated with distinct brain networks, we employed the FC of the two seeds with 7 predefined neural networks. Compared to sham, FC between dlPFC and the frontoparietal network was increased in the active group after treatment ($t(49) = 2.801$, $p = 0.007$, *Cohen's d* = 0.802), whereas FC between dmPFC and the limbic network was decreased ($t(49) = -2.406$, $p = 0.020$, *Cohen's d* = −0.687). No other FC with networks was observed. Functional annotation analysis additionally revealed that both neural circuits identified were indeed associated with major depression. The neural circuits of dlPFC were also linked to cognitive control, whereas the neural circuits of the dmPFC were enriched in the functions relating to emotion processing (Figure S4; Table S6).

Having identified the dissociable altered connections underlying the dual-site protocol, we further investigated predictive therapeutic capacity of these neural circuits. Participants with lower FC between dlPFC and left latOFC ($r = -0.479$, $p = 0.016$) and higher FC between dmPFC and sgACC ($r = 0.440$, $p = 0.026$) at baseline were more likely to benefit from our protocol, indicated by superior long-term therapeutic effects (Figure 4; Table S7).

DISCUSSION

We introduced an innovative accelerated dual-site rTMS protocol (CamFAST-P: Cambridge Fudan Accelerated Sequential TMS-Pudong) in a double-blind RCT sequentially targeting left dlPFC and dmPFC, as an intervention for MDD. All participants exhibited good tolerability. As there were baseline differences in sex distribution between groups, we conducted analyses including controlling for sex as a covariate of no interest and we showed that sex did not influence our clinical results. The active group demonstrated superior efficacy with an average MADRS reduction of 50.98% and response and remission rates of 57.69% and 38.46%, respectively, and critically with rapid antidepressant improvement, response and remission rate relative to sham-control by day 4. Treatment resulted in lower fatigue and higher apathy scores. We show proof of target engagement with dissociable effects of symptom improvement associated with increased dlPFC-frontoparietal FC implicated in cognitive reward control circuitry,[28] and decreased dmPFC-limbic FC implicated in emotion and depressive symptoms.[29,30] Connectivity between dlPFC and lateral OFC and dmPFC and sgACC further predicted week 4 post-treatment outcomes. Moreover, our protocol showed prolonged efficacy as the open-label follow-up showed over 85% responders at week 4 assessment remained in remission for at least 6-month after treatment. We highlight the potential superiority of our dlPFC-dmPFC rTMS protocol over conventional once-daily dlPFC rTMS treatments, characterized by a reduced number of stimulation pulses and a shorter overall treatment duration. This enhanced efficacy is likely to be facilitated by the distinctive dual-site stimulation setting employed in our protocol.

The large and rapid antidepressant effect may be attributed to several factors. Firstly, the dual-site targeting, including stimulation at dlPFC and dmPFC sequentially, may facilitate the effectiveness of this rTMS treatment. An increasing number of studies have reached a consensus that rTMS should be conceptualized as a network therapy.[31] Stimulation at two brain regions influencing two underlying brain networks might lead to an integrated modulational benefit from both networks. The well-established abnormalities in the central executive network and default-mode network in MDD prompted us to use dlPFC and dmPFC as stimulation sites in our study. Here we demonstrate that the dual-site protocol can modulate the dlPFC-frontoparietal network and the dmPFC-limbic network respectively. Similarly,

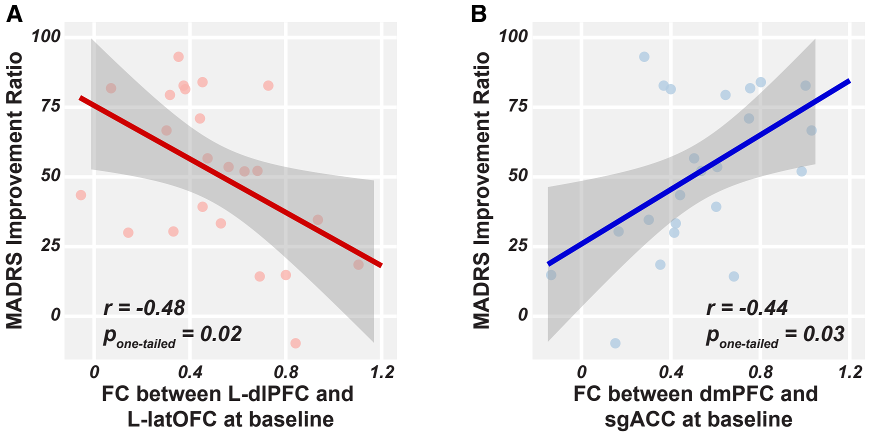

Figure 4. Associations between the long-term therapeutic effect and the specific functional connectivity at baseline

(A) Correlation of left dlPFC-left latOFC FC at baseline and MADRS improvement ratio at week 4.

(B) Correlation of dmPFC-sgACC at baseline and MADRS improvement ratio at week 4. Dots are represented as individual data. Shadows represent 95% confidence interval.

an open-label rTMS study with 10 daily sessions in a small sample of nicotine use disorders demonstrated differential effect as a function of targeting: dlPFC stimulation increased inhibitory capacity and superior medial frontal cortex stimulation reduced craving.[32]

Alternatively, the initial dlPFC stimulation may serve as a "pre-conditioning" stimulation subsequently enhancing effectiveness of dmPFC stimulation, suggesting a "priming effect." Studies using motor evoked potentials (MEP) consistently demonstrate that sequential stimulation may have distinct effects depending on stimulation sequences and intervals.[25] For instance, intermitted iTBS followed by another iTBS enhances the MEP when intervals exceed 15 min presumably mediated by non-homeostatic mechanisms,[33–35] whereas shorter intervals have an opposite effect presumably via homeostatic mechanisms.[34,36] The potential for leveraging the priming effect for rTMS efficacy in MDD has been tested by delivering low-frequency followed by high-frequency stimulations at dlPFC but results are mixed.[3,37,38] The priming effect between two different brain regions in MDD has not been adequately studied yet. Neuroimaging studies have demonstrated that rTMS at dlPFC can influence dmPFC activity.[39,40] Our analysis also reveals a high network overlap between dlPFC and dmPFC, providing the neural basis of priming interaction. Notably, the interval between our two stimulations was less than 5 min, suggesting a potential suppressive effect for the second stimulation exemplified in decreased dmPFC-limbic FC. Therefore, it is plausible that the dlPFC stimulation facilitates the dmPFC network which studies have suggested potentially has a better antidepressant effect.[18,19] Further studies are required to ascertain potential mechanisms underlying this protocol.

The second factor contributing to the substantial antidepressant effect refers to the 50-min inter-session-interval used in our protocol. Previous studies have suggested that the cumulative effect of neural plasticity persists between 50 and 90 min, with intervals less than 40 min yielding more varied results.[13,34,41] This inter-session-interval holds particular significance for accelerated rTMS protocols, as the scheduling of sessions within a defined time frame is largely contingent on this interval.[7] Several early studies using short inter-session-intervals up to 30 min have shown negative clinical outcomes when compared to sham[42–44] or standard groups,[45] while intervals of 50 min or longer seem to have better efficacy.[10,46] We highlight this inter-session-interval in the context of accelerated rTMS protocols.

In addition, the efficacy of our approach may be attributed to the innovative 3-min 20-Hz stimulation sequence. This sequence is safe and well tolerated.[21] Some studies have suggested that 20-Hz stimulation may be more effective than iTBS[47] although further research is needed. One major advantage of our 20-Hz sequence is its ability to deliver 1,200 pulses within the 3-min stimulation duration, doubling the pulse number of the 3.2-min iTBS sequence. The increased number of pulses delivered in a brief time frame may not only enhance effectiveness[10,48] but also expedite the onset of the antidepressant effect, enabling more treatment sessions in a condensed period.

One intriguing observation of our study was a delayed treatment effect, which manifested several weeks after the completion of the treatment sessions. This phenomenon has been observed in multiple rTMS studies not only in depression,[49] but also in other neuropsychiatric disorders.[50] There is evidence indicating that patients may show distinct response trajectories to rTMS.[51] These observations highlight the potential for rTMS to induce long-lasting changes in brain function and symptomatology. And it is also noteworthy that the selection of the endpoint in rTMS studies may be crucial for understanding the full scope of rTMS benefits in clinical practice.

We note that the total pulses used was relatively low at 38,400 in comparison to the SNT (90,000) or the standard FDA-approved 10-Hz protocol (60,000). Furthermore, our treatment spans only four half days, a much shorter duration compared to the 5-day SNT protocol and the traditional 4-week protocol. In addition, the MDD participants in our study are outpatients with moderate symptoms. It has been suggested that individuals with more severe symptoms may derive greater benefits from rTMS,[52,53] while alternative perspectives exist.[10,54,55] When pooling the response and remission rates consistent with the SNT study, we showed that during the 4-week follow-up, 20 out of 26 participants (76.92%) in the active group met response criteria, with 14 (53.85%) meeting remission criteria. In the sham-controlled group, 10 participants (38.46%) responded, and 7 (26.92%) achieved remission. These results indicated a larger proportion of potential responders or remitters given further ongoing treatment. Studies with larger sample sizes and specific experiment designs are required to address these questions.

In the current study, we were unable to access a neuronavigator, leading to the localization of stimulation targets solely through scalp measurements. The dlPFC 5-cm rule target, used in our study, is FDA-approved standard clinical targeting,[56] with superior efficacy compared to various classical approaches.[57] Different dlPFC targets may involve different brain networks,[58] hence targeting various subtypes or symptom dimensions.[14,58,59] Notably, a recent large-scale study has shown equivalent efficacy between MRI-neuronavigated and non-navigated rTMS focusing on dlPFC and anterior insula FC.[60] Instead of personalizing the dlPFC "monotherapy" target, some studies have proposed that other targets (or networks) may enhance the treatment efficacy in addition to the dlPFC treatment.[61–63] Thus, the active debate remains as to whether "personalized monotherapy" or "multiple network targeting" is better for patients. Our protocol uses a scalp-targeting approach with relatively high efficacy, suggesting its potential utility and generalizability in clinical applications.

Limitations of the study

This study has several limitations. Firstly, we were not able to access a sham coil, which limits the effectiveness of blindness. To mitigate this issue, we employed three strategies: (1) comprehensive training for the treatment provider to ensure uniformity and minimize bias; (2) inclusion of only rTMS-naïve participants to avoid experience of any prior sensations or skin contractions related to stimulation; and (3) identification of perceived group assignment before unblinding. Additionally, clinical outcomes revealed only a transient placebo effect in the sham-controlled group, as observed in MADRS, Fatigue Severity Scale (FSS), and AES-C. Therefore, we assert the successful blinding of our study. Secondly, better efficacy may have been achieved by the use of neuronavigator, which can enhance the individualized precision targeting. However, on the other hand, scalp targeting is cost-effective for patients and hospitals that may face budget constraints and cannot afford treatments involving neuronavigators. Thirdly, the course length of the current protocol is only 16 sessions although with 2 sets of stimulation in each session would be 32 sessions, while most other accelerated regimens have delivered 40–50 sessions.[10] Previous studies have shown that a substantial number of responders may require more than 30 sessions to achieve remission.[64] Thus, more rTMS sessions may be warranted to maximum the treatment effect. Fourthly, although a recent study has shown that the dlPFC seed used in our fMRI analyses overlaps with the majority of 5-cm rule defined rTMS targets,[58] it is still inevitable that the 5-cm rule targeting may generate variation in actual target site. Finally, our study lacks a direct comparison with the standard protocol, and the sample size of the current RCT is small. Future research with a larger sample size and comparison with double dlPFC stimulation to match pulse number is indicated.

In summary, we highlight our dual-site rTMS protocol, featuring a relatively small number of pulses and minimal time commitment, while yielding a substantial effect size and a rapid and long-lasting antidepressant onset. This remarkable efficacy may benefit from our dual-site approach that targets dissociable networks. Our findings require confirmation a large-scale study and have potential real-world generalizability and clinical impact.

RESOURCE AVAILABILITY

Lead contact

Requests for further information and resources should be directed to and will be fulfilled by the lead contact, Valerie Voon (vv247@cam.ac.uk).

Materials availability

This study did not generate new, unique reagents.

Data and code availability

- All data reported in this paper will be shared by the lead contact upon request.
- All original code has been deposited at https://github.com/Shitong-Xiang/Dual_Site_TMS_MDD.
- Any additional information required to reanalyze the data reported in this paper is available from the lead contact upon request.

ACKNOWLEDGMENTS

This study was supported by the STI 2030 - Major Projects (grant no. 2021ZD0200407 to V.V.), the National Natural Science Foundation of China (grant nos. 82201670 to Y.-J.Z. and T2250710686 to V.V.), the Clinical Research Project of Shanghai Municipal Commission of Health (grant no. 20244Y0053 to Y.-J.Z.), the Academic Leaders Training Program of Shanghai Pudong New Area Health Commission (grant no. PWRd2024-10 to Y.-J.Z.), the Medical Research Council Senior Clinical Fellowship (grant no. MR/W020408/1 to V.V.), and Tongji University Medicine-X Interdisciplinary Research Initiative (grant no. 2025-0708-ZD- 02 to Y.-J.Z.). We sincerely thank all participants for taking part.

AUTHOR CONTRIBUTIONS

Conceptualization, Y.-J.Z., H.C., W.L., and V.V.; methodology, Y.-J.Z., S.X., W.L., and V.V.; investigation, Y.-J.Z., R.C., Q.D., R.G., Yuan Wang, Y.L., H. L., Yichen Wang, and Y.H.; writing – original draft, Y.-J.Z. and S.X.; writing – review & editing, Y.-J.Z., S.X., W.L., and V.V.; funding acquisition, Y.-J.Z. and V.V.; resources, J.F., W.L., and V.V.; and supervision, J.F., W.L., and V.V.

DECLARATION OF INTERESTS

The authors declare no competing interests.

STAR★METHODS

Detailed methods are provided in the online version of this paper and include the following:

- KEY RESOURCES TABLE
- EXPERIMENTAL MODEL AND STUDY PARTICIPANT DETAILS
  - Eligibility criteria
  - Power analysis
- METHOD DETAILS
  - Randomization and blinding of treatment
  - Procedure and outcome measures
  - Transcranial magnetic stimulation
- QUANTIFICATION AND STATISTICAL ANALYSIS
  - Clinical analysis
  - MRI data acquisition
  - fMRI data preprocessing
  - Overview of fMRI data analysis
  - Seed definition
  - Weighted voxel co-activation network analysis
  - Functional annotation analysis
- ADDITIONAL RESOURCES

CellPress
OPEN ACCESS

SUPPLEMENTAL INFORMATION

Supplemental information can be found online at https://doi.org/10.1016/j.xcrm.2025.102402.

Received: September 29, 2024
Revised: July 30, 2025
Accepted: September 10, 2025
Published: October 1, 2025

REFERENCES

1. Ferrari, A., Santomauro, D., Mantilla, H.A., Shadid, J., Ashbaugh, C., Erskine, H., Charlson, F., Degenhardt, L., Scott, J., McGrath, J., et al. (2022). Global, regional, and national burden of 12 mental disorders in 204 countries and territories, 1990-2019: a systematic analysis for the Global Burden of Disease Study 2019. Lancet Psychiatry *9*, 137–150. https://doi.org/10.1016/S2215-0366(21)00395-3.
2. Li, G., Fife, D., Wang, G., Sheehan, J.J., Bodén, R., Brandt, L., Brenner, P., Reutfors, J., and Dibernardo, A. (2019). All-cause mortality in patients with treatment-resistant depression: a cohort study in the US population. Ann. Gen. Psychiatry *18*, 23. https://doi.org/10.1186/s12991-019-0248-0.
3. Brunoni, A.R., Chaimani, A., Moffa, A.H., Razza, L.B., Gattaz, W.F., Daskalakis, Z.J., and Carvalho, A.F. (2017). Repetitive Transcranial Magnetic Stimulation for the Acute Treatment of Major Depressive Episodes. JAMA Psychiatry *74*, 143–152. https://doi.org/10.1001/jamapsychiatry.2016.3644.
4. Schutter, D.J.L.G. (2009). Antidepressant efficacy of high-frequency transcranial magnetic stimulation over the left dorsolateral prefrontal cortex in double-blind sham-controlled designs: a meta-analysis. Psychol. Med. *39*, 65–75. https://doi.org/10.1017/S0033291708003462.
5. Slotema, C.W., Blom, J.D., Hoek, H.W., and Sommer, I.E.C. (2010). Should we expand the toolbox of psychiatric treatment methods to include Repetitive Transcranial Magnetic Stimulation (rTMS)? A meta-analysis of the efficacy of rTMS in psychiatric disorders. J. Clin. Psychiatry *71*, 873–884. https://doi.org/10.4088/JCP.08m04872gre.
6. Chen, L., Klooster, D.C.W., Tik, M., Thomas, E.H.X., Downar, J., Fitzgerald, P.B., Williams, N.R., and Baeken, C. (2023). Accelerated Repetitive Transcranial Magnetic Stimulation to Treat Major Depression: The Past, Present, and Future. Harv. Rev. Psychiatry *31*, 142–161. https://doi.org/10.1097/hrp.0000000000000364.
7. Sonmez, A.I., Camsari, D.D., Nandakumar, A.L., Voort, J.L.V., Kung, S., Lewis, C.P., and Croarkin, P.E. (2019). Accelerated TMS for Depression: A systematic review and meta-analysis. Psychiatry Res. *273*, 770–781. https://doi.org/10.1016/j.psychres.2018.12.041.
8. Williams, N.R., Sudheimer, K.D., Bentzley, B.S., Pannu, J., Stimpson, K. H., Duvio, D., Cherian, K., Hawkins, J., Scherrer, K.H., Vyssoki, B., et al. (2018). High-dose spaced theta-burst TMS as a rapid-acting antidepressant in highly refractory depression. Brain *141*, e18. https://doi.org/10.1093/brain/awx379.
9. Caulfield, K.A., Fleischmann, H.H., George, M.S., and McTeague, L.M. (2022). A transdiagnostic review of safety, efficacy, and parameter space in accelerated transcranial magnetic stimulation. J. Psychiatr. Res. *152*, 384–396. https://doi.org/10.1016/j.jpsychires.2022.06.038.
10. Cole, E.J., Phillips, A.L., Bentzley, B.S., Stimpson, K.H., Nejad, R., Barmak, F., Veerapal, C., Khan, N., Cherian, K., Felber, E., et al. (2022). Stanford Neuromodulation Therapy (SNT): A Double-Blind Randomized Controlled Trial. Am. J. Psychiatry *179*, 132–141. https://doi.org/10.1176/appi.ajp.2021.20101429.
11. Cole, E.J., Stimpson, K.H., Bentzley, B.S., Gulser, M., Cherian, K., Tischler, C., Nejad, R., Pankow, H., Choi, E., Aaron, H., et al. (2020). Stanford Accelerated Intelligent Neuromodulation Therapy for Treatment-Resistant Depression. Am. J. Psychiatry *177*, 716–726. https://doi.org/10.1176/appi.ajp.2019.19070720.
12. Smolen, P., Zhang, Y., and Byrne, J.H. (2016). The right time to learn: mechanisms and optimization of spaced learning. Nat. Rev. Neurosci. *17*, 77–88. https://doi.org/10.1038/nrn.2015.18.
13. Kramar, E.A., Babayan, A.H., Gavin, C.F., Cox, C.D., Jafari, M., Gall, C.M., Rumbaugh, G., and Lynch, G. (2012). Synaptic evidence for the efficacy of spaced learning. Proc. Natl. Acad. Sci. USA *109*, 5121–5126. https://doi.org/10.1073/pnas.1120700109.
14. Drysdale, A.T., Grosenick, L., Downar, J., Dunlop, K., Mansouri, F., Meng, Y., Fetcho, R.N., Zebley, B., Oathes, D.J., Etkin, A., et al. (2017). Resting-state connectivity biomarkers define neurophysiological subtypes of depression. Nat. Med. *23*, 28–38. https://doi.org/10.1038/nm.4246.
15. Bora, E., Fornito, A., Pantelis, C., and Yücel, M. (2012). Gray matter abnormalities in Major Depressive Disorder: a meta-analysis of voxel based morphometry studies. J. Affect. Disord. *138*, 9–18. https://doi.org/10.1016/j.jad.2011.03.049.
16. Li, C.T., Wang, S.J., Hirvonen, J., Hsieh, J.C., Bai, Y.M., Hong, C.J., Liou, Y.J., and Su, T.P. (2010). Antidepressant mechanism of add-on repetitive transcranial magnetic stimulation in medication-resistant depression using cerebral glucose metabolism. J. Affect. Disord. *127*, 219–229. https://doi.org/10.1016/j.jad.2010.05.028.
17. Stefurak, T., Mikulis, D., Mayberg, H., Lang, A.E., Hevenor, S., Pahapill, P., Saint-Cyr, J., and Lozano, A. (2003). Deep brain stimulation for Parkinson's disease dissociates mood and motor circuits: a functional MRI case study. Mov. Disord. *18*, 1508–1516. https://doi.org/10.1002/mds.10593.
18. Sheline, Y.I., Price, J.L., Yan, Z., and Mintun, M.A. (2010). Resting-state functional MRI in depression unmasks increased connectivity between networks via the dorsal nexus. Proc. Natl. Acad. Sci. USA *107*, 11020–11025. https://doi.org/10.1073/pnas.1000446107.
19. Downar, J., and Daskalakis, Z.J. (2013). New targets for rTMS in depression: a review of convergent evidence. Brain Stimul. *6*, 231–240. https://doi.org/10.1016/j.brs.2012.08.006.
20. Downar, J., Geraci, J., Salomons, T.V., Dunlop, K., Wheeler, S., McAndrews, M.P., Bakker, N., Blumberger, D.M., Daskalakis, Z.J., Kennedy, S.H., et al. (2014). Anhedonia and reward-circuit connectivity distinguish nonresponders from responders to dorsomedial prefrontal repetitive transcranial magnetic stimulation in major depression. Biol. Psychiatry *76*, 176–185. https://doi.org/10.1016/j.biopsych.2013.10.026.
21. Miron, J.P., Feffer, K., Cash, R.F.H., Derakhshan, D., Kim, J.M.S., Fettes, P., Giacobbe, P., Blumberger, D.M., Daskalakis, Z.J., and Downar, J. (2019). Safety, tolerability and effectiveness of a novel 20 Hz rTMS protocol targeting dorsomedial prefrontal cortex in major depression: An open-label case series. Brain Stimul. *12*, 1319–1321. https://doi.org/10.1016/j.brs.2019.06.020.
22. Bakker, N., Shahab, S., Giacobbe, P., Blumberger, D.M., Daskalakis, Z.J., Kennedy, S.H., and Downar, J. (2015). rTMS of the dorsomedial prefrontal cortex for major depression: safety, tolerability, effectiveness, and outcome predictors for 10 Hz versus intermittent theta-burst stimulation. Brain Stimul. *8*, 208–215. https://doi.org/10.1016/j.brs.2014.11.002.
23. Salomons, T.V., Dunlop, K., Kennedy, S.H., Flint, A., Geraci, J., Giacobbe, P., and Downar, J. (2014). Resting-state cortico-thalamic-striatal connectivity predicts response to dorsomedial prefrontal rTMS in major depressive disorder. Neuropsychopharmacology *39*, 488–498. https://doi.org/10.1038/npp.2013.222.
24. Berlim, M.T., Van den Eynde, F., and Daskalakis, Z.J. (2013). A systematic review and meta-analysis on the efficacy and acceptability of bilateral repetitive transcranial magnetic stimulation (rTMS) for treating major depression. Psychol. Med. *43*, 2245–2254. https://doi.org/10.1017/S0033291712002802.
25. Hassanzahraee, M., Zoghi, M., and Jaberzadeh, S. (2018). How different priming stimulations affect the corticospinal excitability induced by noninvasive brain stimulation techniques: a systematic review and meta-analysis. Rev. Neurosci. *29*, 883–899. https://doi.org/10.1515/revneuro-2017-0111.

26. Sliwinska, M.W., Elson, R., and Pitcher, D. (2020). Dual-site TMS demonstrates causal functional connectivity between the left and right posterior temporal sulci during facial expression recognition. Brain Stimul. *13*, 1008–1013. https://doi.org/10.1016/j.brs.2020.04.011.

27. Chen, L., Hudaib, A.R., Hoy, K.E., and Fitzgerald, P.B. (2020). Efficacy, efficiency and safety of high-frequency repetitive transcranial magnetic stimulation applied more than once a day in depression: A systematic review. J. Affect. Disord. *277*, 986–996. https://doi.org/10.1016/j.jad.2020.09.035.

28. Cheng, W., Rolls, E.T., Qiu, J., Liu, W., Tang, Y., Huang, C.C., Wang, X., Zhang, J., Lin, W., Zheng, L., et al. (2016). Medial reward and lateral non-reward orbitofrontal cortex circuits change in opposite directions in depression. Brain *139*, 3296–3309. https://doi.org/10.1093/brain/aww255.

29. Northoff, G. (2016). How do resting state changes in depression translate into psychopathological symptoms? From 'Spatiotemporal correspondence' to 'Spatiotemporal Psychopathology. Curr. Opin. Psychiatr. *29*, 18–24. https://doi.org/10.1097/yco.0000000000000222.

30. Drevets, W.C., Savitz, J., and Trimble, M. (2008). The subgenual anterior cingulate cortex in mood disorders. CNS Spectr. *13*, 663–681. https://doi.org/10.1017/s1092852900013754.

31. Siddiqi, S.H., Kandala, S., Hacker, C.D., Trapp, N.T., Leuthardt, E.C., Carter, A.R., and Brody, D.L. (2023). Individualized precision targeting of dorsal attention and default mode networks with rTMS in traumatic brain injury-associated depression. Sci. Rep. *13*, 4052. https://doi.org/10.1038/s41598-022-21905-x.

32. Chang, D., Zhang, J., Peng, W., Shen, Z., Gao, X., Du, Y., Ge, Q., Song, D., Shang, Y., and Wang, Z. (2018). Smoking Cessation With 20 Hz Repetitive Transcranial Magnetic Stimulation (rTMS) Applied to Two Brain Regions: A Pilot Study. Front. Hum. Neurosci. *12*, 344. https://doi.org/10.3389/fnhum.2018.00344.

33. Opie, G.M., Vosnakis, E., Ridding, M.C., Ziemann, U., and Semmler, J.G. (2017). Priming theta burst stimulation enhances motor cortex plasticity in young but not old adults. Brain Stimul. *10*, 298–304. https://doi.org/10.1016/j.brs.2017.01.003.

34. Tse, N.Y., Goldsworthy, M.R., Ridding, M.C., Coxon, J.P., Fitzgerald, P.B., Fornito, A., and Rogasch, N.C. (2018). The effect of stimulation interval on plasticity following repeated blocks of intermittent theta burst stimulation. Sci. Rep. *8*, 8526. https://doi.org/10.1038/s41598-018-26791-w.

35. Mastroeni, C., Bergmann, T.O., Rizzo, V., Ritter, C., Klein, C., Pohlmann, I., Brueggemann, N., Quartarone, A., and Siebner, H.R. (2013). Brain-derived neurotrophic factor–a major player in stimulation-induced homeostatic metaplasticity of human motor cortex? PLoS One *8*, e57957. https://doi.org/10.1371/journal.pone.0057957.

36. Gamboa, O.L., Antal, A., Moliadze, V., and Paulus, W. (2010). Simply longer is not better: reversal of theta burst after-effect with prolonged stimulation. Exp. Brain Res. *204*, 181–187. https://doi.org/10.1007/s00221-010-2293-4.

37. Fitzgerald, P.B., Hoy, K.E., Singh, A., Gunewardene, R., Slack, C., Ibrahim, S., Hall, P.J., and Daskalakis, Z.J. (2013). Equivalent beneficial effects of unilateral and bilateral prefrontal cortex transcranial magnetic stimulation in a large randomized trial in treatment-resistant major depression. Int. J. Neuropsychopharmacol. *16*, 1975–1984. https://doi.org/10.1017/s1461145713000369.

38. Mutz, J., Vipulananthan, V., Carter, B., Hurlemann, R., Fu, C.H.Y., and Young, A.H. (2019). Comparative efficacy and acceptability of non-surgical brain stimulation for the acute treatment of major depressive episodes in adults: systematic review and network meta-analysis. BMJ *364*, l1079. https://doi.org/10.1136/bmj.l1079.

39. Paus, T., Castro-Alamancos, M.A., and Petrides, M. (2001). Cortico-cortical connectivity of the human mid-dorsolateral frontal cortex and its modulation by repetitive transcranial magnetic stimulation. Eur. J. Neurosci. *14*, 1405–1411. https://doi.org/10.1046/j.0953-816x.2001.01757.x.

40. Liston, C., Chen, A.C., Zebley, B.D., Drysdale, A.T., Gordon, R., Leuchter, B., Voss, H.U., Casey, B.J., Etkin, A., and Dubin, M.J. (2014). Default mode network mechanisms of transcranial magnetic stimulation in depression. Biol. Psychiatry *76*, 517–526. https://doi.org/10.1016/j.biopsych.2014.01.023.

41. Nettekoven, C., Volz, L.J., Kutscha, M., Pool, E.M., Rehme, A.K., Eickhoff, S.B., Fink, G.R., and Grefkes, C. (2014). Dose-dependent effects of theta burst rTMS on cortical excitability and resting-state connectivity of the human motor system. J. Neurosci. *34*, 6849–6859. https://doi.org/10.1523/JNEUROSCI.4993-13.2014.

42. Duprat, R., Desmyter, S., Rudi, D.R., van Heeringen, K., Van den Abbeele, D., Tandt, H., Bakic, J., Pourtois, G., Dedoncker, J., Vervaet, M., et al. (2016). Accelerated intermittent theta burst stimulation treatment in medication-resistant major depression: A fast road to remission? J. Affect. Disord. *200*, 6–14. https://doi.org/10.1016/j.jad.2016.04.015.

43. Baeken, C., Vanderhasselt, M.A., Remue, J., Herremans, S., Vanderbruggen, N., Zeeuws, D., Santermans, L., and De Raedt, R. (2013). Intensive HF-rTMS treatment in refractory medication-resistant unipolar depressed patients. J. Affect. Disord. *151*, 625–631. https://doi.org/10.1016/j.jad.2013.07.008.

44. Desmyter, S., Duprat, R., Baeken, C., Van Autreve, S., Audenaert, K., and van Heeringen, K. (2016). Accelerated Intermittent Theta Burst Stimulation for Suicide Risk in Therapy-Resistant Depressed Patients: A Randomized, Sham-Controlled Trial. Front. Hum. Neurosci. *10*, 480. https://doi.org/10.3389/fnhum.2016.00480.

45. Fitzgerald, P.B., Hoy, K.E., Elliot, D., Susan McQueen, R.N., Wambeek, L. E., and Daskalakis, Z.J. (2018). Accelerated repetitive transcranial magnetic stimulation in the treatment of depression. Neuropsychopharmacology *43*, 1565–1572. https://doi.org/10.1038/s41386-018-0009-9.

46. Loo, C.K., Mitchell, P.B., McFarquhar, T.F., Malhi, G.S., and Sachdev, P.S. (2007). A sham-controlled trial of the efficacy and safety of twice-daily rTMS in major depression. Psychol. Med. *37*, 341–349. https://doi.org/10.1017/S0033291706009597.

47. Andre-Obadia, N., Magnin, M., and Garcia-Larrea, L. (2021). Theta-burst versus 20 Hz repetitive transcranial magnetic stimulation in neuropathic pain: A head-to-head comparison. Clin. Neurophysiol. *132*, 2702–2710. https://doi.org/10.1016/j.clinph.2021.05.022.

48. Li, C.-T., Chen, M.-H., Juan, C.-H., Huang, H.-H., Chen, L.-F., Hsieh, J.-C., Tu, P.-C., Bai, Y.-M., Tsai, S.-J., Lee, Y.-C., and Su, T.-P. (2014). Efficacy of prefrontal theta-burst stimulation in refractory depression: a randomized sham-controlled study. Brain *137*, 2088–2098. https://doi.org/10.1093/brain/awu109.

49. Chen, S.-T., Shaw-Ji, C., Chun-Hung, C., Hsin-Chi, T., and Lin, C.C.H. (2013). Superior antidepressant effect occurring 1 month after rTMS: add-on rTMS for subjects with medication-resistant depression. Neuropsychiatr. Dis. Treat. *397*, 397. https://doi.org/10.2147/ndt.s40466.

50. Li, Z., Yin, M., Lyu, X.L., Zhang, L.L., Du, X.D., and Hung, G.C.L. (2016). Delayed effect of repetitive transcranial magnetic stimulation (rTMS) on negative symptoms of schizophrenia: Findings from a randomized controlled trial. Psychiatry Res. *240*, 333–335. https://doi.org/10.1016/j.psychres.2016.04.046.

51. Kaster, T.S., Downar, J., Vila-Rodriguez, F., Thorpe, K.E., Feffer, K., Noda, Y., Giacobbe, P., Knyahnytska, Y., Kennedy, S.H., Lam, R.W., et al. (2019). Trajectories of Response to Dorsolateral Prefrontal rTMS in Major Depression: A THREE-D Study. Am. J. Psychiatry *176*, 367–375. https://doi.org/10.1176/appi.ajp.2018.18091096.

52. Berlim, M.T., van den Eynde, F., Tovar-Perdomo, S., and Daskalakis, Z.J. (2014). Response, remission and drop-out rates following high-frequency repetitive transcranial magnetic stimulation (rTMS) for treating major depression: a systematic review and meta-analysis of randomized, double-blind and sham-controlled trials. Psychol. Med. *44*, 225–239. https://doi.org/10.1017/S0033291713000512.

53. Sigrist, C., Vöckel, J., MacMaster, F.P., Farzan, F., Croarkin, P.E., Galletly, C., Kaess, M., Bender, S., and Koenig, J. (2022). Transcranial magnetic

CellPress
OPEN ACCESS

stimulation in the treatment of adolescent depression: a systematic review and meta-analysis of aggregated and individual-patient data from uncontrolled studies. Eur. Child Adolesc. Psychiatr. *31*, 1501–1525. https://doi.org/10.1007/s00787-022-02021-7.

54. Grammer, G.G., Kuhle, A.R., Clark, C.C., Dretsch, M.N., Williams, K.A., and Cole, J.T. (2015). Severity of Depression Predicts Remission Rates Using Transcranial Magnetic Stimulation. Front. Psychiatr. *6*, 114. https://doi.org/10.3389/fpsyt.2015.00114.

55. Fitzgerald, P.B., Hoy, K.E., Anderson, R.J., and Daskalakis, Z.J. (2016). A Study of the Pattern of Response to rTMS Treatment in Depression. Depress. Anxiety *33*, 746–753. https://doi.org/10.1002/da.22503.

56. Lefaucheur, J.P., Aleman, A., Baeken, C., Benninger, D.H., Brunelin, J., Di Lazzaro, V., Filipović, S.R., Grefkes, C., Hasan, A., Hummel, F.C., et al. (2020). Evidence-based guidelines on the therapeutic use of repetitive transcranial magnetic stimulation (rTMS): An update (2014-2018). Clin. Neurophysiol. *131*, 474–528. https://doi.org/10.1016/j.clinph.2019.11.002.

57. Fitzgerald, P.B. (2021). Targeting repetitive transcranial magnetic stimulation in depression: do we really know what we are stimulating and how best to do it? Brain Stimul. *14*, 730–736. https://doi.org/10.1016/j.brs.2021.04.018.

58. Cardenas, V.A., Bhat, J.V., Horwege, A.M., Ehrlich, T.J., Lavacot, J., Mathalon, D.H., Glover, G.H., Roach, B.J., Badran, B.W., Forman, S.D., et al. (2022). Anatomical and fMRI-network comparison of multiple DLPFC targeting strategies for repetitive transcranial magnetic stimulation treatment of depression. Brain Stimul. *15*, 63–72. https://doi.org/10.1016/j.brs.2021.11.008.

59. Siddiqi, S.H., Taylor, S.F., Cooke, D., Pascual-Leone, A., George, M.S., and Fox, M.D. (2020). Distinct Symptom-Specific Treatment Targets for Circuit-Based Neuromodulation. Am. J. Psychiatry *177*, 435–446. https://doi.org/10.1176/appi.ajp.2019.19090915.

60. Morriss, R., Briley, P.M., Webster, L., Abdelghani, M., Barber, S., Bates, P., Brookes, C., Hall, B., Ingram, L., Kurkar, M., et al. (2024). Connectivity-guided intermittent theta burst versus repetitive transcranial magnetic stimulation for treatment-resistant depression: a randomized controlled trial. Nat. Med. *30*, 403–413. https://doi.org/10.1038/s41591-023-02764-z.

61. Tadayonnejad, R., Citrenbaum, C., Ngo, T.D.P., Corlier, J., Wilke, S.A., Slan, A., Distler, M.G., Hoftman, G., Adelekun, A.E., Leuchter, M.K., et al. (2023). Right lateral orbitofrontal cortex inhibitory transcranial magnetic stimulation for treatment of refractory mood and depression. Brain Stimul. *16*, 1374–1376. https://doi.org/10.1016/j.brs.2023.09.011.

62. Prentice, A., Kolken, Y., Tuttle, C., van Neijenhof, J., Pitch, R., van Oostrom, I., Kruiver, V., Downar, J., Sack, A.T., Arns, M., and van der Vinne, N. (2023). 1Hz right orbitofrontal TMS benefits depressed patients unresponsive to dorsolateral prefrontal cortex TMS. Brain Stimul. *16*, 1572–1575. https://doi.org/10.1016/j.brs.2023.10.005.

63. Feffer, K., Fettes, P., Giacobbe, P., Daskalakis, Z.J., Blumberger, D.M., and Downar, J. (2018). 1Hz rTMS of the right orbitofrontal cortex for major depression: Safety, tolerability and clinical outcomes. Eur. Neuropsychopharmacol. *28*, 109–117. https://doi.org/10.1016/j.euroneuro.2017.11.011.

64. Stubbeman, W.F., Zarrabi, B., Bastea, S., Ragland, V., and Khairkhah, R. (2018). Bilateral neuronavigated 20Hz theta burst TMS for treatment refractory depression: An open label study. Brain Stimul. *11*, 953–955. https://doi.org/10.1016/j.brs.2018.04.012.

65. Faul, F., Erdfelder, E., Lang, A.G., and Buchner, A. (2007). G*Power 3: a flexible statistical power analysis program for the social, behavioral, and biomedical sciences. Behav. Res. Methods *39*, 175–191. https://doi.org/10.3758/bf03193146.

66. Bech, P., Gram, L.F., Dein, E., Jacobsen, O., Vitger, J., and Bolwig, T.G. (1975). Quantitative rating of depressive states. Acta Psychiatr. Scand. *51*, 161–170. https://doi.org/10.1111/j.1600-0447.1975.tb00002.x.

67. Dunlop, B.W., Parikh, S.V., Rothschild, A.J., Thase, M.E., DeBattista, C., Conway, C.R., Forester, B.P., Mondimore, F.M., Shelton, R.C., Macaluso, M., et al. (2019). Comparing sensitivity to change using the 6-item versus the 17-item Hamilton depression rating scale in the GUIDED randomized controlled trial. BMC Psychiatry *19*, 420. https://doi.org/10.1186/s12888-019-2410-2.

68. Esteban, O., Markiewicz, C.J., Blair, R.W., Moodie, C.A., Isik, A.I., Erramuzpe, A., Kent, J.D., Goncalves, M., DuPre, E., Snyder, M., et al. (2019). fMRIPrep: a robust preprocessing pipeline for functional MRI. Nat. Methods *16*, 111–116. https://doi.org/10.1038/s41592-018-0235-4.

69. Pruim, R.H.R., Mennes, M., van Rooij, D., Llera, A., Buitelaar, J.K., and Beckmann, C.F. (2015). ICA-AROMA: A robust ICA-based strategy for removing motion artifacts from fMRI data. Neuroimage *112*, 267–277. https://doi.org/10.1016/j.neuroimage.2015.02.064.

70. Yeo, B.T.T., Krienen, F.M., Sepulcre, J., Sabuncu, M.R., Lashkari, D., Hollinshead, M., Roffman, J.L., Smoller, J.W., Zöllei, L., Polimeni, J.R., et al. (2011). The organization of the human cerebral cortex estimated by intrinsic functional connectivity. J. Neurophysiol. *106*, 1125–1165. https://doi.org/10.1152/jn.00338.2011.

71. Dunlop, K., Woodside, B., Olmsted, M., Colton, P., Giacobbe, P., and Downar, J. (2016). Reductions in Cortico-Striatal Hyperconnectivity Accompany Successful Treatment of Obsessive-Compulsive Disorder with Dorsomedial Prefrontal rTMS. Neuropsychopharmacology *41*, 1395–1403. https://doi.org/10.1038/npp.2015.292.

72. Mir-Moghtadaei, A., Giacobbe, P., Daskalakis, Z.J., Blumberger, D.M., and Downar, J. (2016). Validation of a 25% Nasion-Inion Heuristic for Locating the Dorsomedial Prefrontal Cortex for Repetitive Transcranial Magnetic Stimulation. Brain Stimul. *9*, 793–795. https://doi.org/10.1016/j.brs.2016.05.010.

73. Van Essen, D.C., Ugurbil, K., Auerbach, E., Barch, D., Behrens, T.E.J., Bucholz, R., Chang, A., Chen, L., Corbetta, M., Curtiss, S.W., et al. (2012). The Human Connectome Project: a data acquisition perspective. Neuroimage *62*, 2222–2231. https://doi.org/10.1016/j.neuroimage.2012.02.018.

74. Langfelder, P., and Horvath, S. (2008). WGCNA: an R package for weighted correlation network analysis. BMC Bioinf. *9*, 559. https://doi.org/10.1186/1471-2105-9-559.

75. Mumford, J.A., Horvath, S., Oldham, M.C., Langfelder, P., Geschwind, D.H., and Poldrack, R.A. (2010). Detecting network modules in fMRI time series: a weighted network analysis approach. Neuroimage *52*, 1465–1476. https://doi.org/10.1016/j.neuroimage.2010.05.047.

76. Langfelder, P., and Horvath, S. (2007). Eigengene networks for studying the relationships between co-expression modules. BMC Syst. Biol. *1*, 54. https://doi.org/10.1186/1752-0509-1-54.

77. Jia, T., Ing, A., Quinlan, E.B., Tay, N., Luo, Q., Francesca, B., Banaschewski, T., Barker, G.J., Bokde, A.L.W., Bromberg, U., et al. (2020). Neurobehavioural characterisation and stratification of reinforcement-related behaviour. Nat. Hum. Behav. *4*, 544–558. https://doi.org/10.1038/s41562-020-0846-5.

78. Liu, Z., Rolls, E.T., Liu, Z., Zhang, K., Yang, M., Du, J., Gong, W., Cheng, W., Dai, F., Wang, H., et al. (2019). Brain annotation toolbox: exploring the functional and genetic associations of neuroimaging results. Bioinformatics *35*, 3771–3778. https://doi.org/10.1093/bioinformatics/btz128.

STAR★METHODS

KEY RESOURCES TABLE

REAGENT or RESOURCE	SOURCE	IDENTIFIER
Software and algorithms		
MATLAB R2018b	The MathWorks	https://matlab.mathworks.com/
SPSS 26	IBM	https://www.ibm.com/analytics/spss-statistics-software
R 3.6.0	Open source	https://www.r-project.org/
fMRIPrep 20.2.7	NiPreps	https://fmriprep.org/en/stable/
Fsl v6.0.7	Oxford	https://fsl.fmrib.ox.ac.uk/fsl/
Afni 24.3.06	NIH	https://afni.nimh.nih.gov/

EXPERIMENTAL MODEL AND STUDY PARTICIPANT DETAILS

Eligibility criteria

Inclusion criteria included: 1) people aged 18 to 55 years; 2) a primary diagnosis of depression based on Diagnostic and Statistical Manual of Mental Disorders Fifth Edition (DSM5) criteria; 3) MADRS score $\geq$20; 4) lack of clinical response or intolerance to at least 1 antidepressant treatment of adequate dose and duration in the current episode; 5) On stable antidepressant medication regimen for at least 4 weeks prior to TMS therapy and agree to keep the medication status stable during the treatment and the 4-week follow-up period; and 6) informed consent form must be signed indicating that the participant understands the purpose of and procedures required for the study and is willing to participate in the study.

Exclusion criteria included: (1) A current or prior diagnosis of a major psychiatric disorder (e.g., substance use disorder, psychosis, bipolar disorder, anorexia, obsessive compulsive disorder, schizophrenia) or MDD with psychotic features, bipolar or related disorders; (2) Acute suicidal or violent behavior or history of suicide attempt within the last 4 weeks; (3) Other severe or unstable medical condition (e.g., major surgery or stroke) or neurological diseases (e.g., Parkinson' disease) or evidence of cognitive impairments that could interfere with the conduct of the current study, or pose any unacceptable risk to the participant; (4) History of other non-invasive neuromodulation treatment such as Electroconvulsive Therapy (ECT), Modified Electroconvulsive Therapy, rTMS, transcranial Electric Stimulation or Vagus Nerve Stimulation; (5) Presence of a specific contraindication for TMS/MRI (e.g., history of seizures, pacemaker or metallic implant); (6) Participant with claustrophobia; (7) Participant who is pregnant, breast-feeding, or planning to become pregnant while enrolled in this study; and (8) Unable to comply with study visit schedule and timeline.

Participants were required to be stable on medication for at least 4 weeks prior to study entry with no change in medication throughout the study.

Power analysis

Sample size was calculated for an estimated effect size of *Cohen's d* = 0.8 for change in MADRS between active and sham-controlled groups. We set the type I error rate of 5% (two-sided) and a power of 80%. As a result, 26 participants are required to reach the statistical power. Considering a dropout rate of 10%, we aimed to recruit 29 participants in each group and 58 in total. The sample size calculation was performed by G*Power 3.1.[65]

METHOD DETAILS

This double-blinded, single-centre RCT was registered with ChiCTR (www.chictr.org.cn, ChiCTR2100046042) and was conducted from June 2021 to December 2022, with follow-up completed by May 2023. This study is approved by the Ethics committee of the Zhongshan hospital (B2020-204R). All participants provided written informed consent before enrollment in the study.

Randomization and blinding of treatment

Participants were assigned in a 1:1 ratio to receive either active or sham rTMS treatment by using a computer-generated randomization sequence. The assessor and participants were blinded to the rTMS conditions. Only TMS naive participants were included to avoid the prior experience of the stimulation sensation. Treatment providers were not blinded and the assessor and treatment providers were separate individuals. Participants were instructed not to discuss the treatment with other participants, or discuss their guess of group assignment with the assessor before unblinding. If multiple participants were at the clinic at the same time, they were seated in separate waiting areas. During the treatment, participants were seated facing away from the TMS device and the coil. Before unblinding (after the week-4 assessment), the participants as well as the assessor were asked to guess the group assignment.

Procedure and outcome measures

A graphic overview of the study is presented in Figure 1 and the timeline for treatment and assessments is provided in Table S1. The assessment included clinical-rated scales, self-rated scales and MRI scanning. The clinical-rated scales included the MADRS (as the primary outcome measure), the CGI (Busner, 2007) and the AES-C. We assessed three sub-scales of CGI: severity of illness (SI, 0-not ill, 7-extremely ill), global improvement (GI, 1-very much improved, 7-very much worse) and efficacy index (EI, 0-no clinical efficacy and severe side effect, 4-significant efficacy and no side effect). The self-rated scales included the BDI, the SHAPS, the STAI and the FSS. All scales were assessed at baseline, at the last day after all treatment sessions and at 2 and 4 weeks after treatment. For MRI, T1-weighted imaging and rsfMRI were scanned at baseline and rsfMRI was also scanned two days after the treatment.

During the 4-day treatment, a simplified 6-question Hamilton Depression Rating Scale (HAMD-6)[66,67] was used to assess acute symptoms after each day of treatment. After unblinding, the sham-controlled group were offered active treatment.

To assess long-term efficacy, all placebo patients were offered the dual stimulation treatment at 4 weeks and we further conducted a 6-month open-label follow-up assessing MADRS scores for all responders at week-4, including participants in the active group and the sham-treated group.

Transcranial magnetic stimulation

TMS was conducted using the pulsed magnetic stimulation device (M-100 Ultimate, Shenzhen Yingchi Technology Co., Ltd, Shenzhen, China) and a liquid cooled flat figure-8 coil (77 mm diameter, model BY90A) was used for both targets. Resting motor threshold (RMT) was obtained from left motor cortex identified as the minimum intensity required to evoke at least 5 out of 10 MEP (MEPs) > 0.05 mV in amplitude via electrodes attached to the right abductor pollicis brevis muscle. The rTMS intensity was 100% individual hand RMT for both dlPFC and dmPFC targets. The left dlPFC was defined as the site 5 cm anterior to the M1. The dmPFC was localized at 25% of the midline distance from nasion to inion.[21] Sham rTMS was carried out with the coil tilted 90° perpendicular to the skull.

We used a 20-Hz sequence for both sites in which the rTMS was on for 2 s and off for 4 s and repeated for 30 times, resulting in a 3-min sequence with 1200 pulses in total, which has been shown comparable efficacy and safety to traditional protocols in a previous large sample study including data from 2528 sessions in 123 MDD individuals.[21] Participants received one 3-min dlPFC stimulation followed by one 3-min dmPFC stimulation with a 5-min interval with inter-session interval at least 50 min. Sixteen sessions of rTMS were administered for 4 consecutive days with 4 sessions per day. Each participant received 19,200 pulses over each stimulation site.

QUANTIFICATION AND STATISTICAL ANALYSIS

Clinical analysis

Clinical assessments were conducted by the same psychiatrist to prevent any potential bias. Our primary outcome was the MADRS score focusing on the week-4 follow-up as an endpoint of the RCT. Clinical response was defined as MADRS reduction ≥50% from baseline and remission as MADRS ≤10. Responders and remitters were identified if the participants met the specific criteria at week-4. Statistical analyses were conducted by mixed-measures ANOVA using SPSS 26 (IBM, Armonk, N.Y.). Asterisks represent significant level: *$p < 0.05$; **$p < 0.01$; ***$p < 0.001$.

MRI data acquisition

MRI data were collected using a Siemens 3T Prisma scanner and a 64-channel RF coil at Fudan University. Whole-brain T1-weighted imaging was conducted with an MPRAGE sequence: TR 3000 ms, TE 2.56 ms, flip angle 7°, matrix size 320 × 320, Field-of-view 256 mm, voxel size 0.8 mm × 0.8 mm, slice thickness 0.8 mm. Functional images were acquired with an Echo-Planar Imaging sequence: TR 2000 ms, TE 30 ms, flip angle 75°, matrix size 102 × 102 with parallel imaging acceleration (GRAPPA) 2, field-of-view 204 mm, voxel size 2 mm × 2 mm, slice thickness 2 mm with a distance factor of 10%, multi-band 2. The number of slices was 56. Phase encoding direction was posterior to anterior.

fMRI data preprocessing

All functional images were preprocessed with the same procedure by suggested protocols from fMRIPrep[68] (version 20.2.7). Briefly, a reference volume and its skull-stripped version were firstly generated. A B0-nonuniformity map (i.e., fieldmap) was estimated based on a phase-difference map calculated with a dual-echo gradient-recall echo sequence. This fieldmap was then co-registered to the target reference and a corrected functional reference was calculated for a more accurate co-registration with the corresponding anatomical reference. Co-registration was configured with 9° of freedom to account for distortions remaining in the functional reference. Head-motion and slice-time were corrected, generating a preprocessed BOLD time-series. These time-series were then co-registered into the MNI standard space. Automatic removal of motion artifacts using independent component analysis (ICA-AROMA[69]) was performed on the preprocessed BOLD on MNI space time-series after removal of non-steady state volumes and spatial smoothing with an isotropic, Gaussian kernel of 6mm full-width half-maximum. Finally, the preprocessed data were re-sampled at a resolution of 3*3*3 mm^3.

Overview of fMRI data analysis

Using dlPFC and dmPFC as seeds, we assessed whole brain FC and 7 predefined functional neural networks,[70] including visual, somatomotor, dorsal attention, ventral attention, limbic, frontoparietal and default mode networks. We initially validated the spatial similarity of dlPFC and dmPFC FC maps using the Human Connectome Project (HCP) database with both correction and the Jaccard index. Then, independent t-tests between the active and sham-controlled groups, employing voxel-wise FC changes for each seed, were conducted. Accordingly, the weighted voxel co-activation network analysis (WVCNA) was used to extract ROIs with *Cohen's* $d > 0.8$. utilizing the HCP database. ROIs were then selected for further analysis based on the following criteria: 1) significant differences in the active group compared to the sham-controlled group as a function of treatment; 2) significant changes before and after the stimulation within the active group. ROIs meeting these criteria for either stimulation target were used to calculate the correlation between FC changes and improvement ratios of MADRS at week. Lastly, an exploratory predictive analysis was conducted by correlating baseline FCs with MADRS improvement ratios, aiming to identify which FC could predict treatment efficacy.

Seed definition

The dlPFC seed was defined by using the HCP label 8Av as it has been demonstrated in a recent study that this region of interest (ROI) overlaps with the majority of 5cm rule defined rTMS targets.[58] The dmPFC seed was defined by creating an ROI using the coordinate of (x0, y+60, z+60) in the Talairach stereotaxic space as the center with a sphere radius of 6mm. This coordinate corresponds to the approximately 25% of the distance from nasion to inion[71,72] and has been used in previous dmPFC stimulation.[20,22,23]

Weighted voxel co-activation network analysis

A total of 1096 participants' preprocessed resting-state fMRI data were collected from the HCP.[73] The voxel-wise seed-based FC was calculated to identify functional modules, which were functionally connected to the left dlPFC and/or dmPFC. Given the high spatial similarity between the FC networks of the two targets, consensus network analysis in the WVCNA was implemented through the R package WGCNA.[74–76] To ensure homogeneity within the functional modules, only 2095 voxels with significant alterative FC to at least one of the seed regions (i.e., effect sizes of the differences between the active group and sham-controlled group greater than 0.8) were included in subsequent analysis. The final dataset used for clustering included 1071 participants for the left dlPFC-seed-based networks and 1031 participants for the dmPFC-seed-based networks after removing null data and outliers. We transferred most parameters as default settings from previous studies,[77] except for the soft-threshold parameters, which were set to four based on the scale-free topology criteria. The stabilities of the generated modules were assessed through bootstrapping.

Functional annotation analysis

Functional annotation analysis of the dominate alterative FC was performed with brain annotation toolbox.[78] Specifically, it can determine which functions are associated with those alterative neural circuits. The functional annotation (i.e., the 217 function terms) was selected from the Neurosynth database. For an FC link, the co-activation ratio for a term was firstly calculated. For a functional network consisting of serval FC links, its extent of activation for a specific functional term was defined as the mean co-activation ratio. The significance was assessed using non-parametric permutation tests. In each permutation run, the same number of non-overlapping regions consisting of the same number of voxels as those in the resulting list from the background were randomly selects to computed the mean co-activation ratio. Total 10000 times were repeated to obtain the distribution of the null hypothesis.

ADDITIONAL RESOURCES

This study is registered with ChiCTR (ChiCTR2100046042). URL: www.chictr.org.cn.

Cell Reports Medicine, Volume *6*

Supplemental information

A sequential dual-site repetitive transcranial magnetic stimulation for major depressive disorder: A randomized clinical trial

Yi-Jie Zhao, Shitong Xiang, Ruiqin Chen, Qiong Ding, Ruijie Geng, Yuan Wang, Yuanyuan Li, Haibin Li, Yichen Wang, Hailun Cui, Ying Huang, Jianfeng Feng, Wenjuan Liu, and Valerie Voon

Supplementary information for:

A Sequential Dual-Site Repetitive Transcranial Magnetic Stimulation for Major Depressive disorders: A Randomized Clinical Trial

Yi-Jie Zhao; Shitong Xiang; Ruiqin Chen; Qiong Ding; Ruijie Geng; Yuan Wang; Yuanyuan Li; Haibin Li; Yichen Wang; Hailun Cui; Ying Huang; Jianfeng Feng; Wenjuan Liu; Valerie Voon

Contents

Supplementary Tables **2**

Table S1 Timeline for treatment and assessments. Related to Figure 1. 2

Table S2 Descriptive statistics of MADRS scores. Related to Figure 2. 2

Table S3 27 clusters identified by the WVCNA. Related to Figure 3. 3

Table S4 Statistics of Seed-Based Functional Connectivity (FC) with 27 clusters. Related to Figure 3. 3

Table S5 Statistics of Seed-Based Functional Connectivity (FC) Change Associated with MADRS improvement Ratio. Related to Figure 3. 4

Table S6 Functional Annotation Analysis Results. Related to Figure 3 and Figure S4. 5

Table S7 Statistics of Baseline Seed-Based Functional Connectivity (FC) Associated with MADRS improvement Ratio. Related to Figure 4. 6

Supplementary Figures **7**

Figure S1. Results of secondary outcomes. Related to Figure 2. 7

Figure S2. Individual MADRS scores for long-term follow-up. Related to Figure 2. 7

Figure S3. High spatial similarity between the patterns of functional connectivity (FC) networks with the left dlPFC and dmPFC. Related to Figure 3. 8

Figure S4. Function annotation analysis results. Related to Figure 3 and Table S6 8

Supplementary Tables

Table S1 Timeline for treatment and assessments. Related to Figure 1.

	Baseline	Day 1	Day 2	Day 3	Day 4	Day 6	week-2 (Day 18)	week-4 (Day 32)
rTMS		•	•	•	•			
HAMD-6		•	•	•	•			
Clinician-rated scales	•				•		•	•
Self-rated scales	•				•		•	•
T1-weighted	•							
rsfMRI	•					•		

Abbreviations: rTMS, repetitive Transcranial Magnetic Stimulation; HAMD-6, Hamilton Depression Rating Scale (6-question version); rsfMRI, resting-state functional Magnetic Resonance Imaging.

Table S2 Descriptive statistics of MADRS scores. Related to Figure 2.

		**Active group(n=26)**				**Sham-controlled group(n=26)**			
		Mean	SD	95% CI		Mean	SD	95% CI	
				Lower Bound	Upper Bound			Lower Bound	Upper Bound
**MARDS score**	Baseline	27.42	3.82	25.92	28.92	26.15	4.25	24.55	27.75
	Treat end	16.04	6.99	13.34	18.74	20.85	5.97	18.55	23.15
	Two week	12.38	7.13	9.68	15.08	17.92	7.38	15.12	20.72
	Four week	13.50	8.04	10.4	16.6	21.31	8.38	18.11	24.51
**MARDS improvement ratio**	Treat end	0.42	0.25	0.32	0.51	0.19	0.25	0.09	0.29
	Two week	0.54	0.27	0.44	0.64	0.30	0.30	0.19	0.41
	Four week	0.51	0.27	0.41	0.61	0.17	0.33	0.04	0.30

*MARDS = Montgomery-Åsberg Depression Rating Scale, SD = Standard Deviation, 95% CI =95% Confidence Interval

Table S3 27 clusters identified by the WVCNA. Related to Figure 3.

Cluster label	Peak Point in MNI Space			Voxel Size	Brain Region Label
	x	y	z		
Cluster1	42	-17	51	407	Right pre/postcentral cortex
Cluster2	-53	7	29	213	Left inferior frontal cortex
Cluster3	48	-33	45	188	Right supramarginal gyrus
Cluster4	46	8	34	144	Right inferior frontal cortex
Cluster5	-50	20	9	121	Left inferior frontal cortex
Cluster6	-36	-17	62	113	Left postcentral cortex
Cluster7	-1	6	63	112	Supplementary motor cortex
Cluster8	40	-47	47	101	Right inferior parietal cortex
Cluster9	-44	-39	51	98	Left inferior parietal cortex
Cluster10	25	-24	-17	93	Right amygdala/hippocampus
Cluster11	-4	14	48	90	Middle cingulate cortex
Cluster12	37	-7	60	85	Right superior frontal cortex
Cluster13	-37	-43	43	81	Left inferior parietal cortex
Cluster14	-34	4	58	68	Left dorsolateral prefrontal cortex
Cluster15	-35	51	22	58	Left middle frontal cortex
Cluster16	-16	-8	-16	56	Left amygdala/hippocampus
Cluster17	-32	-54	49	52	Left superior parietal cortex
Cluster18	-61	-48	30	48	Left supramarginal gyrus
Cluster19	5	-3	-8	43	Right ventral thalamus
Cluster20	-51	-22	-28	41	Left inferior temporal cortex
Cluster21	37	38	28	41	Right middle frontal cortex
Cluster22	-48	38	-6	31	Left lateral orbitofrontal cortex
Cluster23	36	15	-34	29	Right temporal pole
Cluster24	52	-16	-26	23	Right inferior temporal cortex
Cluster25	39	45	-11	23	Right lateral orbitofrontal cortex
Cluster26	39	53	7	21	Right middle frontal cortex
Cluster27	0	38	-8	20	Subgenual anterior cingulate cortex

Table S4 Statistics of Seed-Based Functional Connectivity (FC) with 27 clusters. Related to Figure 3.

Cluster label	dlPFC FC					dmPFC FC				
	FC Change (Active vs Sham)			Post vs pre (Active)		FC Change (Active vs Sham)			Post vs pre (Active)	
	t	*p*	*Cohen' d*	t	*p*	*t*	*p*	*Cohen'd*	*t*	*p*
Cluster1	**3.316**	**0.002**	**1.011**	1.614	0.107	1.381	0.174	0.421	0.437	0.662
Cluster2	**3.108**	**0.003**	**0.948**	1.802	0.072	1.999	0.052	0.610	0.572	0.568
Cluster3	**2.830**	**0.007**	**0.863**	1.364	0.173	1.870	0.068	0.570	0.438	0.662
**Cluster4**	**3.555**	**0.001**	**1.084**	**2.039**	**0.042**	1.842	0.072	0.562	0.743	0.458
**Cluster5**	**2.765**	**0.008**	**0.843**	**2.035**	**0.042**	**2.429**	**0.019**	**0.741**	1.478	0.140
Cluster6	**2.756**	**0.009**	**0.841**	1.462	0.144	1.126	0.266	0.344	0.252	0.801
Cluster7	**3.409**	**0.001**	**1.040**	1.714	0.087	1.811	0.077	0.552	0.535	0.593
Cluster8	**2.432**	**0.019**	**0.742**	0.656	0.512	1.637	0.109	0.499	0.452	0.651
Cluster9	**2.679**	**0.010**	**0.817**	1.625	0.104	**2.343**	**0.024**	**0.715**	1.029	0.304
**Cluster10**	**-2.395**	**0.021**	**-0.730**	-1.820	0.069	**2.472**	**0.017**	**-0.754**	**-2.752**	**0.006**
Cluster11	**3.150**	**0.003**	**0.961**	1.840	0.066	1.810	0.077	0.552	0.743	0.458
Cluster12	**2.859**	**0.007**	**0.872**	1.005	0.315	1.453	0.153	0.443	0.080	0.936
Cluster13	**2.809**	**0.007**	**0.857**	1.533	0.125	**2.035**	**0.048**	**0.621**	0.512	0.609
Cluster14	**2.398**	**0.021**	**0.731**	1.665	0.096	0.523	0.603	0.160	-0.447	0.655
**Cluster15**	**3.400**	**0.001**	**1.037**	**2.575**	**0.010**	1.360	0.181	0.415	1.163	0.245
**Cluster16**	**-2.971**	**0.005**	**-0.906**	**-2.080**	**0.038**	**4.061**	**0.000**	**-1.239**	**-2.777**	**0.006**
Cluster17	**2.797**	**0.008**	**0.853**	1.865	0.062	1.508	0.139	0.460	1.054	0.292
Cluster18	**3.010**	**0.004**	**0.918**	1.093	0.275	1.786	0.081	0.545	0.850	0.396
**Cluster19**	-1.982	0.054	-0.604	-0.941	0.347	**4.121**	**0.000**	**-1.257**	**-2.540**	**0.011**
**Cluster20**	**-3.371**	**0.002**	**-1.028**	**-2.996**	**0.003**	**3.066**	**0.004**	**-0.935**	**-2.126**	**0.034**
Cluster21	**3.162**	**0.003**	**0.964**	0.981	0.327	1.246	0.219	0.380	0.186	0.852
**Cluster22**	**3.056**	**0.004**	**0.932**	**2.451**	**0.014**	**2.151**	**0.037**	**0.656**	1.823	0.069
**Cluster23**	-1.810	0.077	-0.552	-1.640	0.101	**3.105**	**0.003**	**-0.947**	**-2.160**	**0.031**
**Cluster24**	**-2.893**	**0.006**	**-0.882**	**-2.344**	**0.019**	**2.077**	**0.044**	**-0.634**	-1.660	0.097
**Cluster25**	**3.790**	**0.000**	**1.156**	**1.965**	**0.050**	**2.162**	**0.036**	**0.659**	1.531	0.126
Cluster26	**2.344**	**0.024**	**0.715**	0.835	0.404	1.585	0.120	0.483	1.079	0.281
**Cluster27**	-0.782	0.438	-0.239	-0.398	0.691	**3.726**	**0.001**	**-1.136**	**-2.866**	**0.004**

Table S5 Statistics of Seed-Based Functional Connectivity (FC) Change Associated with MADRS improvement Ratio. Related to Figure 3.

FC	Treatment End		week-4	
	t	*p*	t	*p*
**dlPFC**				
**Right inferior frontal cortex (Cluster4)**	**0.410**	**0.041**	**0.545**	**0.008**
Left inferior frontal cortex (Cluster5)	**0.441**	**0.030**	0.185	0.224
Left middle frontal cortex (Cluster15)	0.044	0.428	0.007	0.488
Left amygdala/hippocampus (Cluster16)	0.235	0.833	-0.010	0.484
Left inferior temporal cortex (Cluster20)	-0.115	0.320	-0.085	0.364
**Left lateral orbitofrontal cortex (Cluster22)**	0.317	0.093	**0.448**	**0.027**
Right inferior temporal cortex (Cluster24)	0.363	0.936	0.333	0.918
**Right lateral orbitofrontal cortex (Cluster25)**	**0.454**	**0.025**	**0.403**	**0.043**
**dmPFC**				
**Right amygdala/hippocampus (Cluster10)**	-0.233	0.169	**-0.433**	**0.032**
**Left amygdala/hippocampus (Cluster16)**	**-0.501**	**0.015**	**-0.470**	**0.021**
Right ventral thalamus (Cluster19)	0.034	0.554	-0.077	0.377
Left inferior temporal cortex (Cluster20)	-0.034	0.445	0.007	0.511
**Right temporal pole (Cluster23)**	-0.307	0.101	**-0.397**	**0.046**
**Subgenual anterior cingulate cortex (Cluster27)**	**-0.491**	**0.016**	**-0.477**	**0.019**

Table S6 Functional Annotation Analysis Results. Related to Figure 3 and Figure S4.

Terms	dlPFC	dmPFC	Terms	dlPFC	dmPFC	Terms	dlPFC	dmPFC
acoustic	0.4166	0.3938	judgment	0.0875	0.1469	recollection	**0.0265**	0.0669
action	0.2364	0.4552	judgment_task	0.0941	0.1421	repetition	0.2807	0.5266
action_observation	0.3362	0.5212	language	**0.0453**	0.3231	repetition_suppression	0.3816	0.3544
adaptation	0.1699	0.4437	language_comprehension	0.3761	0.3469	response_inhibition	**0.0307**	0.4492
affective	0.6278	0.0815	language_network	0.1204	0.5433	response_selection	0.3765	0.3425
anger	0.3337	0.3224	learning_task	0.3973	0.0612	reward	0.2633	0.2452
anticipation	0.4365	0.096	lexical_decision	0.0524	0.3478	reward_anticipation	0.3974	**0.0412**
anxiety	0.4204	**0.0288**	listening	0.2162	0.35	rhythm	0.3272	0.294
anxiety_disorders	0.4324	**0.0188**	major_depression	**0.0321**	**0.0049**	saccades	0.1498	0.2548
arithmetic	0.1833	0.3755	matching_task	0.3523	0.1388	sad	0.5181	**0.0261**
arousal	0.4939	**0.0337**	memory	0.4776	0.0561	salience	0.2735	**0.0173**
attention	0.2816	0.0904	memory_encoding	0.2295	0.4235	schizophrenia	0.3129	0.0954
attention_deficit	0.1185	0.3236	memory_load	**0.0134**	0.2401	secondary_somatosensory	0.4243	0.3926
attention_network	0.5759	0.4296	memory_performance	0.5474	0.0733	selective_attention	0.3884	0.3652
attentional_control	0.1647	0.3551	memory_processes	0.1932	0.4113	self	0.3706	0.0806
auditory_visual	0.4875	0.458	memory_retrieval	**0.0386**	0.0508	self_referential	0.1318	**0.0256**
autism	0.4178	0.0736	memory_task	0.2477	0.0619	self_reported	0.3237	0.318
autobiographical_memory	0.0681	**0.0105**	memory_wm	0.2175	0.3869	semantic	0.0597	0.0976
awareness	0.51	0.1773	mental_imagery	0.3259	0.5073	semantic_information	0.0623	0.1532
bipolar_disorder	0.0994	0.3444	mild_cognitive	0.5394	0.095	semantic_knowledge	0.1295	0.0865
cognition	0.3378	0.1063	monetary_reward	0.4149	0.0782	semantic_memory	0.1367	0.1259
cognitive_control	0.1822	0.321	monitor	0.5109	0.3463	sensation	0.3792	0.3283
cognitive_emotional	0.3735	**0.0443**	mood	0.4313	**0.0262**	sensory_information	0.1588	0.4243
cognitive_functions	0.1413	0.2714	moral	0.1468	0.1341	sensory_modalities	0.2715	0.2341
cognitive_processes	0.171	0.1345	motion	0.291	0.4916	sentence_comprehension	0.0601	0.2292
cognitive_tasks	0.454	0.1774	motivation	0.4507	0.1311	sleep	0.0751	0.4091
communication	0.5419	0.2136	motor_control	0.4563	0.4414	social	0.1415	0.0521
compulsive_disorder	0.1825	0.1301	motor_imagery	0.3011	0.3859	social_cognition	0.3897	0.1223
conscious	0.3338	0.0655	motor_performance	0.3401	0.3135	social_interaction	0.2788	0.0505
control_network	**0.006**	0.4423	motor_response	0.3675	0.4239	somatosensory	0.5477	0.4855
control_processes	0.1258	0.4581	motor_task	0.3882	0.3715	spatial_attention	0.1833	0.4324
decision_making	0.2777	**0.0291**	moving	0.2388	0.4568	speech	0.2713	0.5336
decision_task	0.1169	0.2068	multisensory	0.4145	0.3892	speech_perception	0.4146	0.3998
depression	0.1825	**0.0027**	music	0.3856	0.2521	speech_production	0.4029	0.3824
detection_task	0.206	0.4975	naming	0.4524	0.413	speech_sounds	0.1519	0.2427
discrimination_task	0.5159	0.3533	navigation	0.1462	0.148	spoken	0.4012	0.3719
disgust	0.3786	0.064	negative_affect	0.0507	**0.0409**	stop_signal	0.4037	0.3739
emotion	0.2977	**0.035**	negative_emotional	0.2788	**0.0224**	stress	0.4413	**0.011**
emotion_regulation	**0.0099**	0.0522	neurocognitive	0.2117	0.0944	stroop	**0.0441**	0.3386
emotional_faces	0.4809	**0.0489**	neutral_faces	0.4661	0.0505	stroop_task	**0.0483**	0.2838
emotional_information	0.3957	0.3649	neutral_pictures	0.3069	0.0629	subsequent_memory	**0.043**	0.0588
emotional_responses	0.3132	**0.0116**	neutral_stimuli	0.495	0.0505	suppression	0.6572	0.2827
emotional_stimuli	0.4165	**0.0442**	nociceptive	0.4754	0.4081	sustained_attention	0.3997	0.2364
emotional_valence	0.2917	**0.0177**	nogo	0.2656	0.3983	switch	0.1022	0.3606
empathy	0.2933	0.251	object_recognition	0.4409	0.4283	switching	0.1449	0.3639
encoding_retrieval	0.1859	0.0891	obsessive_compulsive	0.2019	0.1361	syntactic	**0.0361**	0.3103
episode	0.138	0.3339	ongoing	0.2297	0.4546	tapping	0.2983	0.3681
episodic_memory	**0.0406**	0.0744	oral	0.3648	0.3302	target_detection	0.2801	0.4345
executive_control	0.1872	0.4485	orientation	0.3683	0.4154	term_memory	0.1708	0.3655
executive_function	0.1065	0.3223	pain	0.5092	0.1712	theory_mind	0.2058	0.1602
expectancy	0.2149	0.3832	passive_viewing	0.327	0.3181	threatening	0.431	**0.0414**
eye_movements	0.1813	0.3212	percept	0.4968	0.463	touch	0.4446	0.4123
face	0.2185	0.3023	perception	0.5388	0.6207	verbal	0.3101	0.4214
face_recognition	0.3556	0.3463	perceptual	0.4374	0.5938	verbal_fluency	0.254	0.4082
face_stimuli	0.2734	0.255	performance_task	**0.0452**	0.2143	verbal_working	0.231	0.3122
facial_expressions	0.4548	0.0837	personality	0.1787	0.0648	video	0.164	0.5155
fear	0.4775	**0.0209**	phonetic	0.1578	0.3488	visual_attention	0.2115	0.4579
fearful_faces	0.4864	**0.0259**	phonological	0.1257	0.4834	visual_auditory	0.2329	0.4588
finger_movements	0.3906	0.438	picture	0.2427	0.0679	visual_information	0.487	0.4631
fusiform_face	0.3126	0.3069	planning	0.3492	0.4515	visual_motion	0.2907	0.2696
gain	0.3693	0.103	pleasant	0.3125	0.2943	visual_perception	0.3171	0.2981
gambling	0.353	0.0509	pressure	0.3066	0.2701	visual_spatial	0.1302	0.296

goal_directed	0.5107	0.2013	primary_auditory	0.2607	0.246	visual_stimuli	0.2557	0.382
hallucinations	0.1948	0.4605	primary_sensorimotor	0.3532	0.404	visual_stream	0.3453	0.3381
hand_movements	0.4121	0.3937	primary_somatosensory	0.3676	0.3657	visuo_spatial	0.1107	0.2662
happy	0.3804	0.3637	primary_visual	0.3261	0.4118	visuospatial	0.2367	0.4917
hyperactivity_disorder	0.2748	0.0645	reactivity	0.3757	**0.028**	voice	0.1792	0.2297
illusion	0.2955	0.2719	reading	0.1101	0.5151	vulnerability	0.2302	**0.0048**
image	0.4905	0.1982	reasoning	**0.0369**	0.4119	word_recognition	0.3534	0.3324
impulsivity	0.1889	0.0958	recall	**0.0497**	**0.029**	working_memory	0.353	0.5122
incentive	0.2249	0.4252	recognition	**0.0393**	**0.0461**	written	0.0876	0.4169
inhibition	0.2744	0.4286	recognition_memory	0.2278	0.2034	recollection	**0.0265**	0.0669
inhibitory_control	**0.0245**	0.2914	recognition_task	0.451	0.1535	repetition	0.2807	0.5266

Table S7 Statistics of Baseline Seed-Based Functional Connectivity (FC) Associated with MADRS improvement Ratio. Related to Figure 4.

FC	Treatment End		week-4	
	t	*p*	t	*p*
**dlPFC**				
Right inferior frontal cortex (Cluster4)	-0.146	0.269	-0.317	0.087
**Left lateral orbitofrontal cortex (Cluster22)**	**-0.424**	**0.031**	**-0.479**	**0.016**
Right lateral orbitofrontal cortex (Cluster25)	-0.176	0.229	-0.269	0.126
**dmPFC**				
Right amygdala/hippocampus (Cluster10)	0.023	0.462	0.233	0.161
Left amygdala/hippocampus (Cluster16)	**0.391**	**0.044**	0.309	0.093
Right temporal pole (Cluster23)	0.151	0.263	**0.380**	**0.049**
**Subgenual anterior cingulate cortex (Cluster27)**	**0.403**	**0.039**	**0.440**	**0.026**

Supplementary Figures

Figure S1. Results of secondary outcomes. Related to Figure 2.

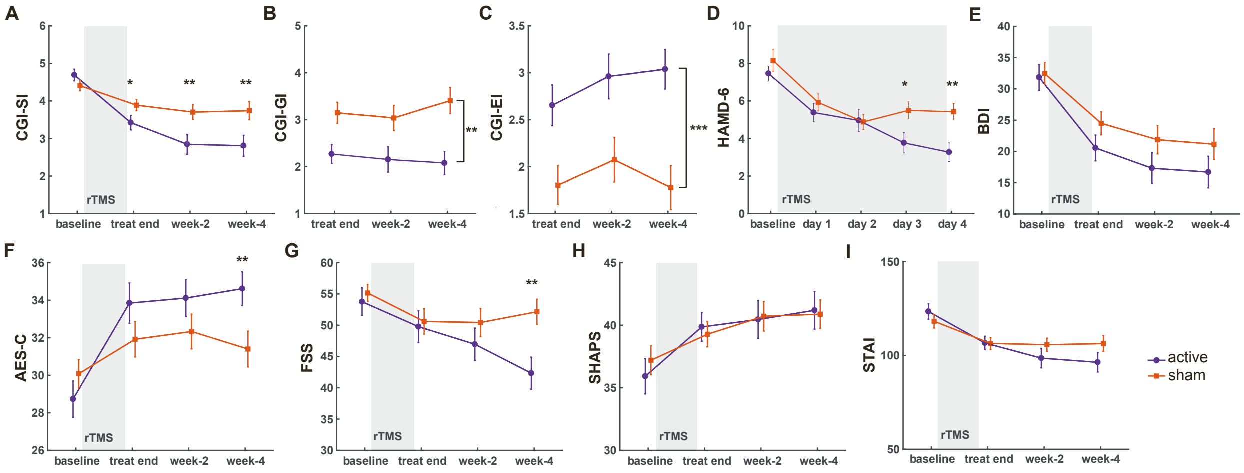

Figure S1. Results of secondary outcomes. CGI: the Clinical Global Impression; SI: severity of illness; GI: global improvement; EI: efficacy index; HAMD: Hamilton Depression Rating Scale; BDI: the Beck Depression Inventory; AES-C the Apathy Evaluation Scale – Clinician rated. (BDI), FSS: the Fatigue Severity Scale; SHAPS: the Snaith Hamilton Pleasure Scale. Data are represented as mean +/- SEM. Statistical analyses were conducted by mixed-measures ANOVA. Significance: $^{*}p$<.05; $^{**}p$<.01; $^{***}p$<.001.

Figure S2. Individual MADRS scores for long-term follow-up. Related to Figure 2.

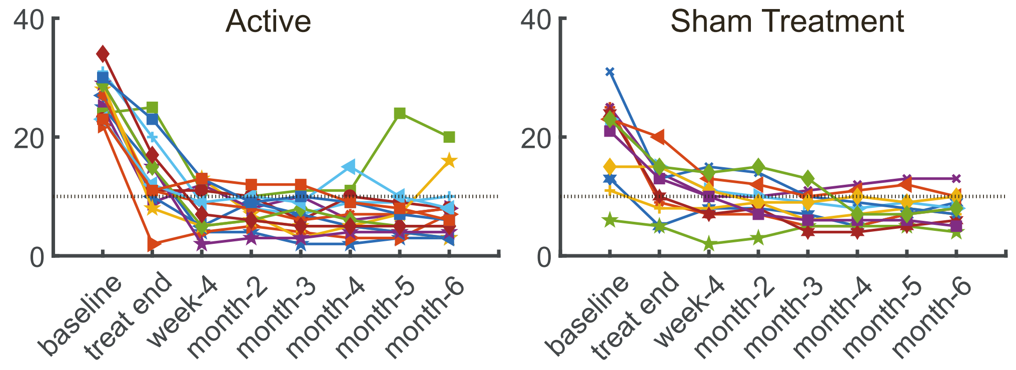

Figure S3. High spatial similarity between the patterns of functional connectivity (FC) networks with the left dlPFC and dmPFC. Related to Figure 3.

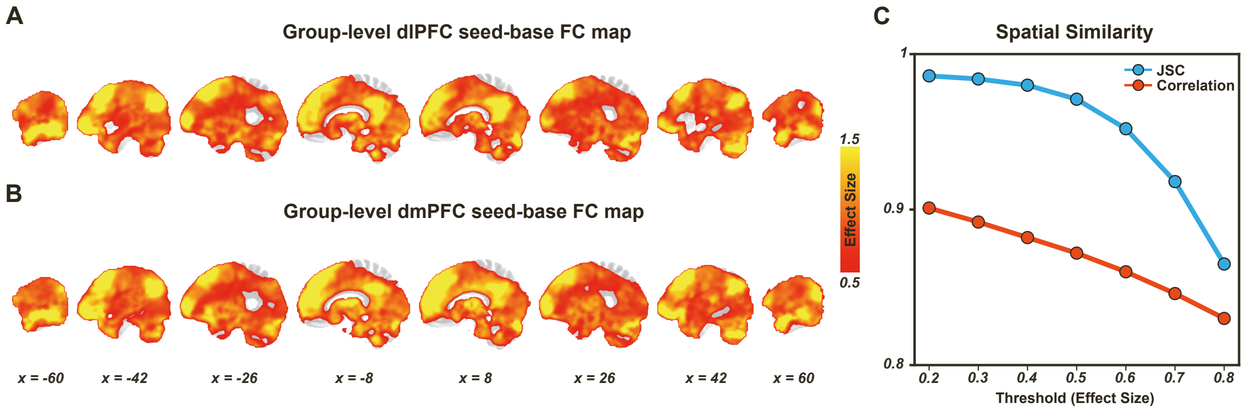

Figure S3. High spatial similarity between the patterns of functional connectivity (FC) networks with the left dlPFC and dmPFC. (a) The seed-based FC network targeting the left dlPFC. **(b)** The seed-based FC network targeting the dmPFC. **(c)** Statistical results of spatial similarity between above two FC networks under different thresholds.

Figure S4. Function annotation analysis results. Related to Figure 3 and Table S6.

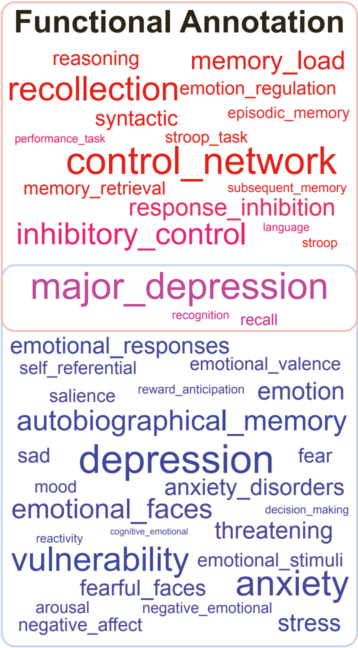

Supplement: Document S2. Article plus supplemental information [file mmc3.pdf]
